# The Diagnostic Value of Image-Based Machine Learning for Osteoporosis: Systematic Review and Meta-Analysis

**DOI:** 10.2196/75965

**Published:** 2026-01-16

**Authors:** Rui Zhao, Haolin Yang, Yangbo Li, Xiaoyun Li, Zhijie Yang, Yanping Lin, Jiachun Huang, Lei Wan, Hongxing Huang

**Affiliations:** 1 The Third Clinical College of Medicine, Guangzhou University of Traditional Chinese Medicine, Guangzhou, China; 2 The Third Affiliated Hospital of Guangzhou University of Chinese Medicine, Guangzhou University of Chinese Medicine, 261 Longxi Avenue, Liwan District, Guangzhou, 510000, China, 86 13922726488

**Keywords:** osteoporosis, machine learning, artificial intelligence, systematic review, diagnostic imaging

## Abstract

**Background:**

Osteoporosis (OP) is projected to be a major issue significantly impacting the well-being of middle-aged and old populations. Machine learning (ML) and deep learning (DL) models developed based on medical imaging have enhanced clinicians’ diagnostic accuracy and work efficiency. However, the diagnostic performance of different types of medical imaging for OP has not been systematically assessed.

**Objective:**

By summarizing related literature, this study aims to elucidate the role of DL models based on different medical imaging modalities in OP detection.

**Methods:**

PubMed, Embase, the Cochrane Library, and Web of Science were systematically searched for studies using ML for the diagnosis of OP based on medical imaging. The final search was conducted on May 16, 2024. The risk of bias in the included studies was assessed using the Quality Assessment of Diagnostic Accuracy Studies-2 tool. A bivariate mixed-effects model was applied to perform meta-analyses of sensitivity (SEN) and specificity (SPC), stratified by imaging modality (x-ray, computed tomography [CT], magnetic resonance imaging [MRI]). In addition, subgroup analyses were carried out based on the type of ML algorithm, the method of validation dataset generation, and the anatomical site of assessment.

**Results:**

A total of 60 studies comprising 66,195 participants were encompassed in this systematic review and meta-analysis. Among these, 22 studies used x-ray imaging, 37 applied CT imaging, and 3 used MRI for ML-based OP diagnosis. For x-ray–based models, the pooled SEN and SPC for studies focusing on the appendicular skeleton were 0.97 (95% CI 0.83‐0.99) and 0.90 (95% CI 0.75‐0.96), respectively. For studies using the mandible as the target site, SEN and SPC were 0.94 (95% CI 0.89‐0.97) and 0.80 (95% CI 0.56‐0.93), respectively. For those focusing on the lumbar spine, the pooled SEN and SPC were 0.87 (95% CI 0.77‐0.93) and 0.82 (95% CI 0.75‐0.87), respectively. For CT-based models, studies targeting the hip joint reported a pooled SEN and SPC of 0.87 (95% CI 0.83‐0.90) and 0.92 (95% CI 0.81‐0.96), respectively. For the thoracic spine, SEN and SPC were 0.91 (95% CI 0.86‐0.94) and 0.94 (95% CI 0.92‐0.95), respectively, while for the lumbar spine, they were 0.91 (95% CI 0.87‐0.94) and 0.92 (95% CI 0.86‐0.95), respectively.

**Conclusions:**

ML based on medical imaging demonstrates high diagnosis accuracy for OP, particularly DL models using x-ray and CT modalities. However, this study included only a limited number of original studies using MRI-based ML, and there remains a lack of adequate external validation across studies, which poses interpretative limitations. Future research should aim to develop artificial intelligence tools with broader applicability and enhanced diagnostic precision.

## Introduction

Osteoporosis (OP), a metabolic disorder, features a systemic reduction in bone mass and impaired bone microarchitecture and elevates the risk of fragility fractures. As the most prevalent chronic metabolic bone disease, it is strongly associated with advancing age, posing significant health threats. However, due to its insidious onset, prolonged disease course, and challenges in treatment, public awareness and attention toward OP prevention and management remain insufficient [1,2]. With the emerging global trend of population aging, OP is projected to become a major issue adversely affecting the quality of life of middle-aged and older people. Epidemiological studies estimate that by 2050, the global population at high risk of fractures will surge to 6.26 million from 1.66 million in 1990 [3]. This escalation imposes immense social pressures and substantial economic burdens on early OP screening, prevention, and treatment.

At present, a variety of diagnostic methods are available for the clinical assessment of OP. Among them, dual-energy x-ray absorptiometry (DXA) for measuring *T* scores recommended by the World Health Organization is regarded as the authoritative and standardized technique [4]. Although DXA is widely used, it is unable to assess whole-body skeletal, fat, and lean mass, which restricts its utility in the routine diagnosis or evaluation of OP [5]. Moreover, due to disparities in socioeconomic development across different regions worldwide, DXA is not accessible in underdeveloped countries and regions. Therefore, some high-risk populations, such as postmenopausal women and older adults, are not detected and untreated. Medical imaging is crucial in clinical diagnosis and treatment. However, the hidden features within imaging techniques including x-rays, computed tomography (CT), and magnetic resonance imaging (MRI) are often overlooked due to low spatial resolution and high contrast resolution [6].

In the 1980s, computer-aided diagnosis (CAD) systems were developed to deeply interpret key features in medical images, providing radiologists with valuable insights into image interpretation [7]. Currently, CAD tools primarily include traditional machine learning (ML) models built on explainable clinical features and deep learning (DL) models developed using pathological or nuclear medicine images. They assist clinicians in disease diagnosis and prognostic prediction. Increasing evidence has demonstrated the utility of CAD in diagnosing conditions such as autism [8], pulmonary embolism [9], breast cancer [10], and bone metastases [11]. DL approaches based on medical imaging have attracted substantial research interest. Against this backdrop, ML models based on various imaging modalities such as x-rays, CT, and MRI have been constructed to diagnose OP [12]. However, the diagnostic performance of various imaging methods in OP is not supported by systematic evidence. This hindered the application of artificial intelligence (AI)–based CAD tools in OP and posed challenges for further systematic development.

Therefore, our study seeks to provide a comprehensive review of DL research in the diagnosis of OP based on medical imaging modalities, including x-ray, CT, and MRI. Furthermore, this study aims to analyze and evaluate the feasibility and accuracy of AI-driven DL in enhancing the screening and diagnostic rates of OP, thereby offering robust support for the prevention and management of the disease.

## Methods

### Study Registration

This study followed the PRISMA (Preferred Reporting Items for Systematic Reviews and Meta-Analyses) guidelines and was prospectively registered on PROSPERO (CRD42024567736).

### Eligibility Criteria

The eligible studies were (1) case-control, cohort, or cross-sectional studies; (2) papers with comprehensively developed image-based DL models for OP diagnosis; and (3) English publications. The following studies were excluded: (1) studies that only developed traditional ML models, (2) those that performed image segmentation without a complete DL model, and (3) those lacking outcome measures for evaluating the DL model’s accuracy. Outcome measures must include at least 1 of the following: c-statistic, sensitivity (SEN), specificity (SPC), accuracy, recall, precision, confusion matrix, *F*_1_-score, or calibration curve.

### Data Sources and Search Strategy

PubMed, Cochrane, Embase, and Web of Science databases were thoroughly retrieved up to May 16, 2024. Both MeSH and free-text terms were used without restrictions on geographic location or study type. The search strategy is detailed in Multimedia Appendix 1.

### Study Selection

The retrieved literature was uploaded to EndNote (Thomson Corporation), and duplicates were ostracized. Titles and abstracts were reviewed to identify potentially eligible studies. Full-text papers were subsequently screened to determine the eligible ones. Two researchers (RZ and HY) independently conducted the literature screening and cross-checked their results. Dissents were addressed by a third researcher (YL).

### Data Extraction

The eligible papers were imported into EndNote, and data extraction was performed. A standard electronic data extraction form was developed beforehand to capture the following information: title, DOI, first author, publication year, author’s country, study type, patient source, OP diagnosis criteria, medical imaging, background population, gender, age, use of image segmentation, number of OP cases, total cases, number of OP and total cases in the training or validation set, validation set generation method, model type, and comparison with clinical practitioners. Data were independently extracted by 2 researchers (RZ and HY), followed by cross-checking. There was a high level of agreement between the 2 reviewers in the screening process (Cohen κ=0.879). In cases of disagreement, a third reviewer (YL) would assist in addressing it.

### Risk of Bias in Studies

The bias of risk in the eligible studies was assessed via Quality Assessment of Diagnostic Accuracy Studies-2, a tool for evaluating the collation risk of bias and clinical applicability of original diagnostic studies [13]. Quality Assessment of Diagnostic Accuracy Studies-2 covers 5 domains: case selection, trials to be evaluated, reference standard, case flow, and progress, with each involving a few specific questions. The answer of “Yes,” “No,” or “Uncertain” corresponds to a low, high, or uncertain risk of bias. The risk of bias was deemed low if all of the landmark questions within a range were answered with “Yes”; if one of the informative questions was answered with “No,” bias may exist, and the evaluators must determine the risk of bias in line with the established guidelines. The risk of bias must be judged by the evaluation authors as per the established criteria. An unclear risk indicated that the studies reported sufficient details. Therefore, evaluators could not make a definitive judgment.

The risk of bias in studies was independently conducted by 2 researchers (RZ and HY), followed by cross-checking. If any dissent arose, a third researcher (YL) would assist in addressing it.

### Synthesis Methods

The meta-analysis was carried out via a bivariate mixed-effects model based on diagnostic 2×2 contingency tables. However, some of the original studies did not provide complete 2×2 diagnostic data. In such cases, the necessary information was derived using SEN, SPC, positive predictive value, negative predictive value, and accuracy, in conjunction with the corresponding sample sizes. The meta-analysis reported pooled estimates of SEN, SPC, positive likelihood ratio (PLR), negative likelihood ratio (NLR), diagnostic odds ratio (DOR), and the summary receiver operating characteristic (SROC) curve along with their corresponding 95% CIs. Publication bias across studies was assessed through Deeks’ funnel plot, while the clinical utility of the predictive models was evaluated via Fagan’s nomogram. Subgroup analyses were performed based on imaging modality (x-ray, CT, and MRI), modeling approach (traditional ML vs DL), and validation strategy. It is important to note that the bivariate mixed-effects model requires a minimum of four 2×2 diagnostic tables. As the ML models based on MRI images only provided 3 such tables, a narrative synthesis was performed for this subgroup instead. A 2-sided *P* value of <.05 denoted statistical significance.

## Results

### Study Registration

**Figure 1.**
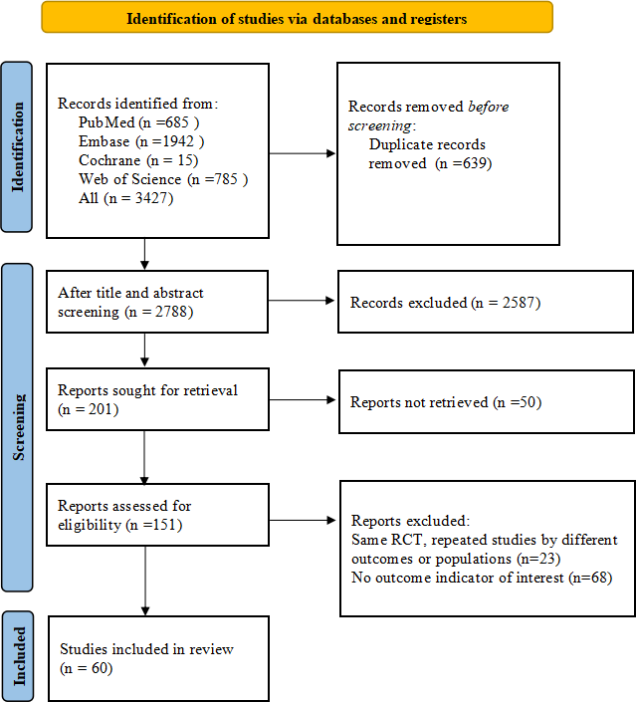
Literature screening process. RCT: randomized controlled trial.

A total of 3427 papers were retrieved, including 685 from PubMed, 15 from Cochrane, 1942 from Embase, and 785 papers from Web of Science. Among them, 639 papers were duplicates and were excluded. After the title and abstract review, 2587 studies unrelated to the study topic were removed. Full texts of the rest were subsequently reviewed. In total, 23 conference abstracts without full-text publications and 68 that did not include medical imaging in the modeling process were ostracized. Ultimately, 60 studies were included in the analysis (Figure 1) [14-73]. This study was conducted in accordance with the PRISMA 2020 checklist (Checklist 1).

### Study Characteristics

Among the 60 studies included in our analysis, 55 were case-control studies [14-21,25-35,37-67,69-73], and 5 were cohort studies [22-24,36,68]. These studies were predominantly published between 2012 and 2024 and involved 66,195 cases. These studies were published in 11 countries, including China (n=27), South Korea (n=10), the United States (n=8), and Japan (n=5) [15-17,20,22,23,26-33,35-48,50,51,53-60,62-73]. A smaller number of studies were from India (n=3), Saudi Arabia (n=2), Jordan (n=1), Latvia (n=1), Malaysia (n=1), Poland (n=1), and Switzerland (n=1) [14,18,19,21,24,25,34,39,52,61]. In total, 57 studies reported patient sources, of which 42 were single-center studies [14,15,17,19-23,26,29,31,32,36-42,47,48,50-60,62,64-67,69-73], 12 were multicenter studies [16,27,28,30,33,34,43-45,61,63,68], and 4 used database sources [24,41,46,49]. In terms of OP diagnosis, 47 studies explicitly provided diagnosis criteria [16,17,20-23,25,27-33,35-42,44,45,47,50-58,60-64,66-73]. Regarding medical imaging, 37 studies developed CT-based imaging models [15,16,18,19,21-23,25,26,29,31-33,38-41,43-45,47,48,50-54,56-58,65,67-69,72,73], 22 developed x-ray–based models [14,17,20,24,27,28,30,34-37,42,46,49,55,59-63,70,71], and 3 focused on MRI-based models [45,64,66]. Concerning the population, 4 studies specifically examined postmenopausal women [16,35,36,52,71], and 1 study focused on men aged 50 years and older [16]. In terms of image processing, 48 studies used manual segmentation techniques to define regions of interest for analysis [14-19,21-23,25-36,38,39,41,42,44,45,47,49,52-57,59-66,68,70-73], while 12 did not define regions of interest [20,24,37,40,43,46,48,50,51,58,67,69]. Regarding the skeletal parts, 33 studies used lumbar vertebrae images [15-17,20,22,23,26,29-32,36,38-41,45,47,48,50,53-55,57,62,64-68,70,71,73], 9 used thoracic vertebrae images [23,25,31,38,44,48,53,55,56], 10 used hip images (including femoral neck, femoral head, and pelvis) [18,21,33,36,57,62,68,70,72], 7 used mandible images [17,36,42,52,60,63,71], and 10 used images of limb bones [14,28,34,35,43,51,58,59,61,69]. Regarding the generation of validation sets, 35 studies adopted random sampling [15-17,20-23,25,27,29-32,36,38,40-42,44,48,50-53,56,57,60-62,64,65,67,69,71,72], 12 used K-fold cross-validation [14,24,34,37,39,46,49,58,59,66,70,73], and 5 applied external validation [28,33,45,54,63]. In total, 9 studies compared their results with the screening results of clinicians [22,25,28,45-47,60,62,65]. In terms of model construction, 32 built DL models [18,21,23-31,33,37-42,44-47,52-56,59-62,66], and 28 constructed ML models [14-17,19,20,22,32,34-36,43,48-51,57,58,63-65,67-73] (Multimedia Appendix 2).

### Risk of Bias in Studies

**Figure 2.**
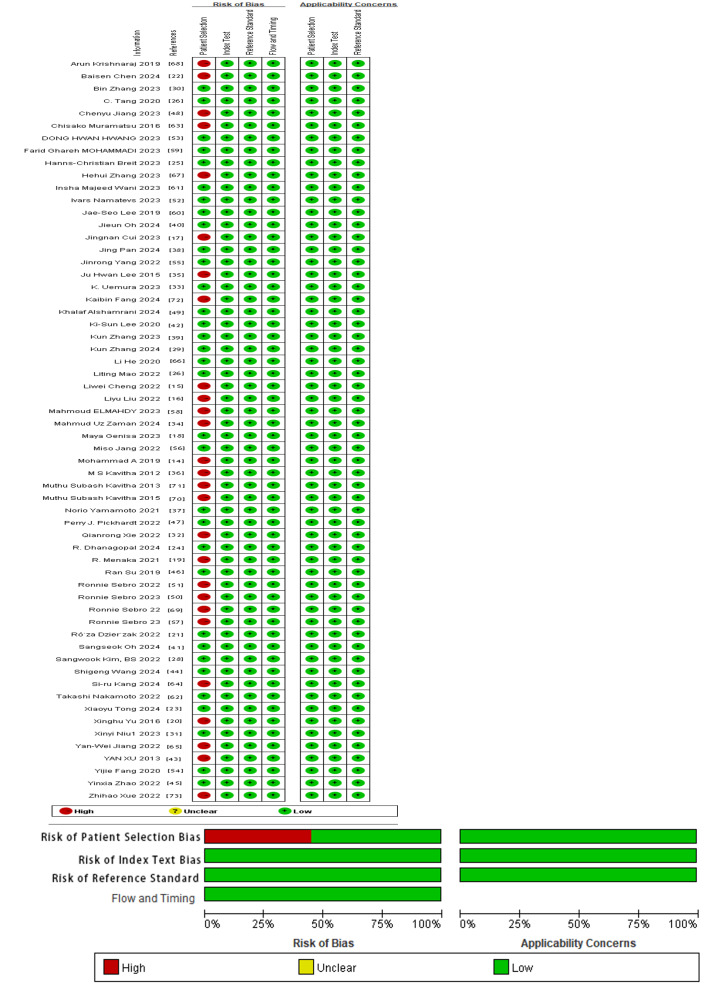
Risk of bias plot [14-73].

In all eligible studies, consecutive cases were included. Although most studies were case-control studies, 32 developed DL models, with variables derived from medical images. Therefore, these studies demonstrated a low risk of bias in case selection. In total, 28 studies applied ML models, where the process of variable generation might be influenced by the case-control study design, thereby leading to a higher risk of bias. Since this research is a meta-analysis of ML, whether or not the reference standards for OP diagnosis are known does not affect the results. Additionally, the criteria for determining positive results were pre-established, indicating that the trials under evaluation posed a low risk of bias. The implementation of a reference standard for OP diagnosis was considered reasonable, thus introducing a low risk of bias. Furthermore, there was a proper time interval between the trial and reference standard, and all patients in a given study followed the same diagnosis rules, with no cases omitted. Therefore, there was a low risk of bias in clinical applicability [14-73] (Figure 2 and Multimedia Appendix 3).

### Meta-Analysis

#### ML Based on X-Ray

##### Synthesized Results

**Figure 3.**
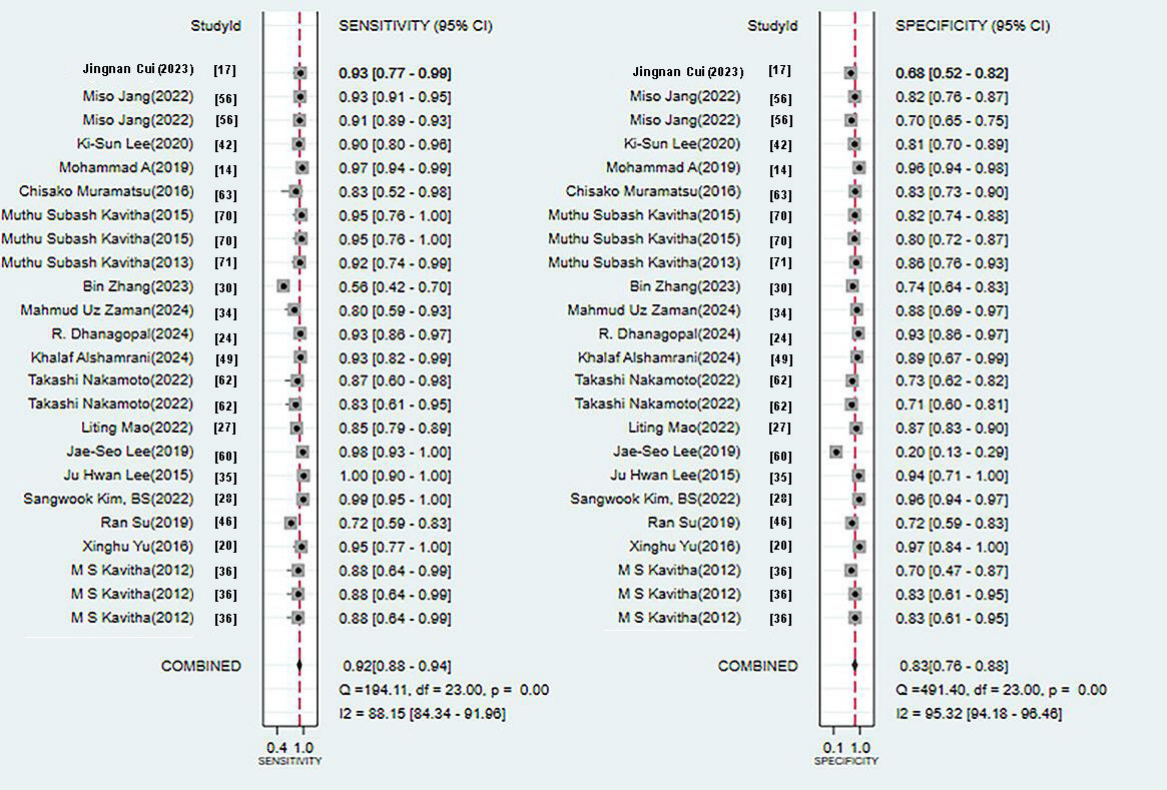
Forest plot of sensitivity and specificity for x-ray–based machine learning for diagnosing osteoporosis [14,17,20,24,27,28,30,34-36,42,46,49,56,60,62,63,70,71].

This validation set comprised 24 diagnostic 4-fold tables, which were used to verify the ML models based on x-rays for OP diagnosis. The results were summarized through the bivariate mixed-effects model. The pooled SEN, SPC, PLR, NLR, DOR, and SROC curves were 0.92 (95% CI 0.88‐0.94), 0.83 (95% CI 0.76‐0.88), 5.4 (95% CI 3.8‐7.6), 0.10 (95% CI 0.07‐0.15), 54 (95% CI 28‐105), and 0.94 (95% CI 0.92‐0.96), respectively (Figures3  4) [14,17,20,24,27,28,30,34-37,42,46,49,55,59-63,70,71]. There was no discernible publication bias in the studies according to Deeks’ funnel plot (Figure 5). In the included study participants, approximately 48.44% (n=6429) had OP. Assuming this as the prior probability, if the ML prediction result was OP, the actual probability of OP was .83. If the ML prediction result was non-OP, the actual probability of non-OP was .92 (Figure 6).

**Figure 4.**
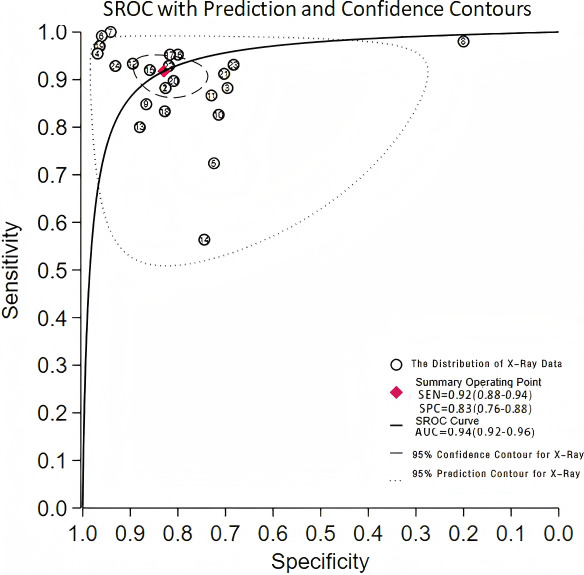
95% Confidence contour for x-ray. AUC: Area underthe curve;SEN: Sensitivity; SPC: Specificity;SROC: Summary receiver operating characteristic.

**Figure 5.**
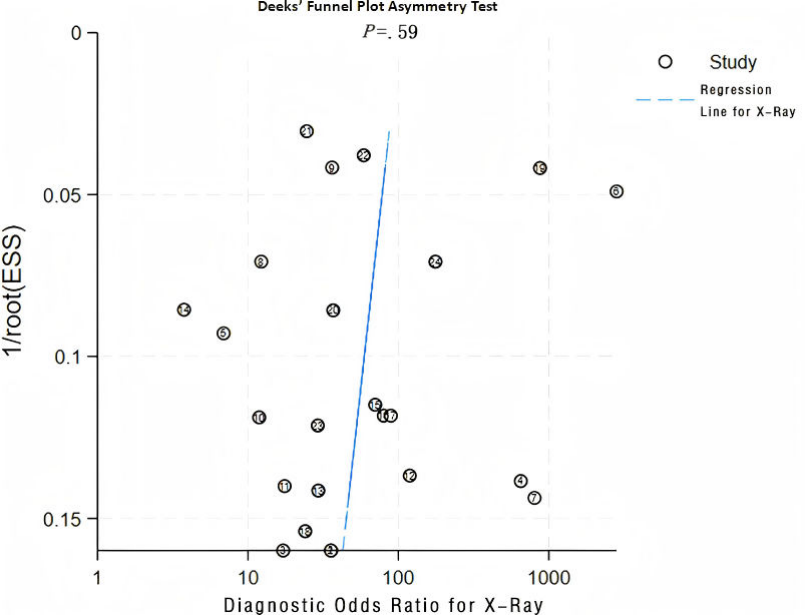
Diagnostic odds ratio for x-ray. ESS:Effective sample size.

**Figure 6.**
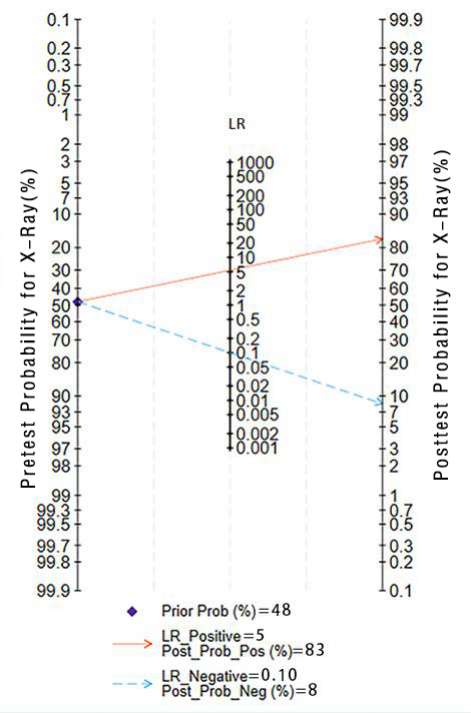
Pretest probability for x-ray. LR:Likelihood ratio.

##### Subgroup Analysis: Types of ML

###### Deep Learning

The validation set included 9 diagnostic 4-fold tables to assess the performance of DL models based on x-ray images for OP diagnosis. The results summarized from the bivariate mixed-effects model showed that SEN, SPC, PLR, NLR, DOR, and the SROC curve were 0.90 (95% CI 0.79‐0.95), 0.79 (95% CI 0.62‐0.89), 4.2 (95% CI 2.2‐8.0), 0.13 (95% CI 0.06‐0.29), 32 (95% CI 9‐107), and 0.92 (95% CI 0.89‐0.94), respectively (Figures S1 and S2 in Multimedia Appendix 4). Deeks’ funnel plot revealed no marked publication bias (Figure S3 in Multimedia Appendix 4). In the encompassed studies, approximately 30% (n=2556) of the participants had OP. Therefore, assuming this as the prior probability, if the result from ML indicated OP, the actual probability of OP was .64. If the ML result indicated non-OP, the actual probability of non-OP was .95 (Figure S4 in Multimedia Appendix 4).

###### Traditional ML

The validation set encompassed 15 diagnostic 4-fold tables for validating the traditional ML models based on x-ray imaging for OP diagnosis. The bivariate mixed-effects model was leveraged. The pooled SEN, SPC, PLR, NLR, DOR, and SROC were 0.93 (95% CI 0.92‐0.95), 0.85 (95% CI 0.79‐0.89), 6.0 (95% CI 4.3‐8.5), 0.08 (95% CI 0.06‐0.10), 78 (95% CI 44‐139), and 0.96 (95% CI 0.94‐0.97), respectively (Figures S5 and S6 in Multimedia Appendix 4). Deeks’ funnel plot showed no significant publication bias in studies (Figure S7 in Multimedia Appendix 4). Approximately 61% (n=3863) of the participants had OP. Therefore, assuming this as the prior probability, if the result from ML indicated OP, the actual probability of OP was .90. If the ML result indicated non-OP, the actual probability of non-OP was .81 (Figure S8 in Multimedia Appendix 4).

### Generation Method of the Validation Set

#### K-Fold Cross-Validation

Among models constructed through x-ray for OP diagnosis, 7 diagnostic 4-fold tables used the K-fold cross-validation to generate the validation set. The results summarized by the bivariate mixed-effects model demonstrated that the SEN, SPC, PLR, NLR, DOR, and SROC curve were 0.90 (95% CI 0.83‐0.95), 0.87 (95% CI 0.79‐0.93), 7.2 (95% CI 4.0‐12.7), 0.11 (95% CI 0.06‐0.21), 64 (95% CI 20‐204), and 0.95 (95% CI 0.93‐0.96), respectively (Figures S9 and S10 in Multimedia Appendix 4). Deeks’ funnel plot did not exhibit significant publication bias (Figure S11 in Multimedia Appendix 4). Among the participants in our included studies, approximately 42% (n=1287) had OP. Therefore, assuming this as the prior probability, if the ML result indicated OP, the actual probability of OP was .84. If the ML result indicated non-OP, the actual probability of non-OP was .92 (Figure S12 in Multimedia Appendix 4).

#### Random Sampling

In total, 14 diagnostic 4-fold tables used the random sampling method to generate the validation set. The results summarized by the bivariate mixed-effects model showed that the pooled SEN, SPC, PLR, NLR, DOR, and SROC curve were 0.90 (95% CI 0.84‐0.93), 0.76 (95% CI 0.67‐0.84), 3.8 (95% CI 2.7‐5.4), 0.14 (95% CI 0.09‐0.20), 28 (95% CI 16‐48), and 0.91 (95% CI 0.88‐0.93), respectively (Figures S13 and S14 in Multimedia Appendix 4). Significant publication bias was not noted in Deeks’ funnel plot (Figure S15 in Multimedia Appendix 4). Among the participants in our included studies, approximately 60% (n=4049) had OP. Therefore, assuming this as the prior probability, if the ML result indicated OP, the actual probability of OP was .85. If the ML result showed non-OP, the actual probability of non-OP was .83 (Figure S16 in Multimedia Appendix 4).

### Examination Parts

#### Limbs

In the OP diagnosis models constructed based on x-rays, 4 diagnostic 4-fold tables focused on the limb bones. The results summarized by the bivariate mixed-effects model showed a SEN of 0.97 (95% CI 0.83‐0.99), SPC of 0.90 (95% CI 0.75‐0.96), PLR of 9.6 (95% CI 3.5‐25.9), NLR of 0.03 (95% CI 0.01‐0.22), DOR of 277 (95% CI 20‐3783), and the SROC curve of 0.98 (95% CI 0.96‐0.99; Figures S17 and S18 in Multimedia Appendix 4). Deeks’ funnel plot did not demonstrate significant publication bias (Figure S19 in Multimedia Appendix 4). In the included study participants, the proportion of OP cases was approximately 19% (n=1114). Assuming this as the prior probability, if the ML result indicated OP, the actual probability of OP was .69. If the ML result indicated non-OP, the actual probability of non-OP was <.001 (Figure S20 in Multimedia Appendix 4).

#### Mandible

In total, 6 diagnostic 4-fold tables focused on the mandible. The bivariate mixed-effects model was used. The pooled SEN, SPC, PLR, NLR, DOR, and SROC were 0.94 (95% CI 0.89‐0.97), 0.80 (95% CI 0.56‐0.93), 4.8 (95% CI 1.9‐12.1), 0.07 (95% CI 0.04‐0.14), 69 (95% CI 20‐241), and 0.96 (95% CI 0.94‐0.97), respectively (Figures S21 and S22 in Multimedia Appendix 4). Deeks’ funnel plot indicated no significant publication bias (Figure S23 in Multimedia Appendix 4). In all included study participants, the proportion of OP cases was approximately 42% (n=1153). Assuming this as the prior probability, if the ML result indicated OP, the actual probability of OP was .78. If the ML result indicated non-OP, the actual probability of non-OP was .95 (Figure S24 in Multimedia Appendix 4).

#### Lumbar Vertebrae

In total, 8 diagnostic 4-fold tables focused on the lumbar vertebrae. The bivariate mixed-effects model was used to summarize data. The pooled SEN, SPC, PLR, NLR, DOR, and SROC were 0.87 (95% CI 0.77‐0.93), 0.82 (95% CI 0.75‐0.87), 4.8 (95% CI 3.4‐6.7), 0.16 (95% CI 0.08‐0.30), 31 (95% CI 12‐77), and 0.90 (95% CI 0.87‐0.92), respectively (Figures S25 and S26 in Multimedia Appendix 4). Significant publication bias was not found in Deeks’ funnel plot (Figure S27 in Multimedia Appendix 4). In the included study participants, the proportion of OP cases was approximately 32% (n=1281). Assuming this as the prior probability, if the ML result indicated OP, the actual probability of having OP was .69. If the ML result indicated non-OP, the actual probability of non-OP was .93 (Figure S28 in Multimedia Appendix 4).

### ML Based on CT

#### Synthesized Results

**Figure 7.**
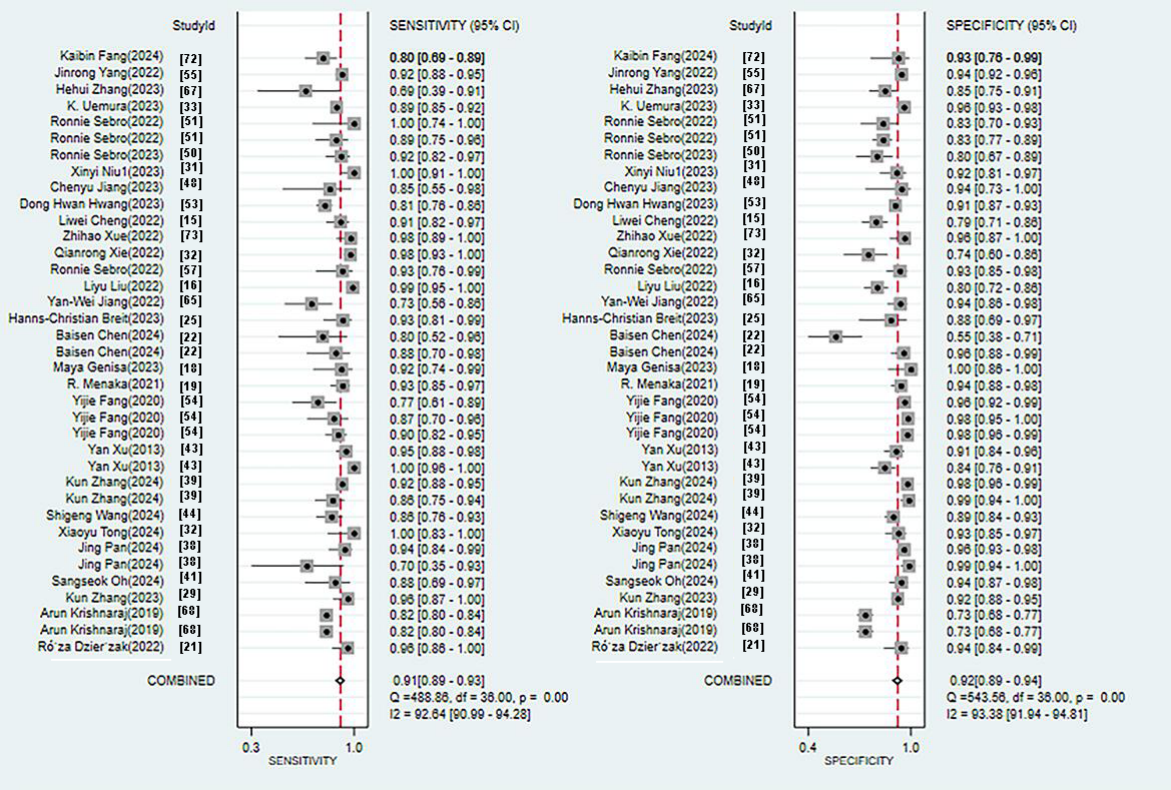
Forestplot of sensitivity and specificity for computed tomography-based machine learning for diagnosing osteoporosis [15,16,18,19,21-23,25,26,29,31-33,38-41,43-45,47,48,50-54,56-58,65,67-69,72,73].

The validation set consisted of 24 diagnostic 4-fold tables for validating CT-based ML models for diagnosing OP. The bivariate mixed-effects model was used to pool data. The pooled SEN, SPC, PLR, NLR, DOR, and SROC were 0.91 (95% CI 0.89‐0.93), 0.92 (95% CI 0.89‐0.94), 11.6 (95% CI 8.5‐9.7), 0.09 (95% CI 0.07‐0.12), 123 (95% CI 80‐90), and 0.97 (95% CI 0.53‐1.00), respectively (Figures7  8) [15,16,18,19,21-23,25,26,29,31-33,38-41,43-45,47,48,50-54,56-58,65,67-69,72,73]. According to Deeks’ funnel plot, there was no significant publication bias (Figure 9). Among the included research participants, the proportion of individuals with OP was approximately 50% (n=10,995). Therefore, assuming this as the prior probability, if the ML models predicted OP, the actual probability of OP was .92. If the ML models predicted no OP, the actual probability of non-OP was .91 (Figure 10).

**Figure 8.**
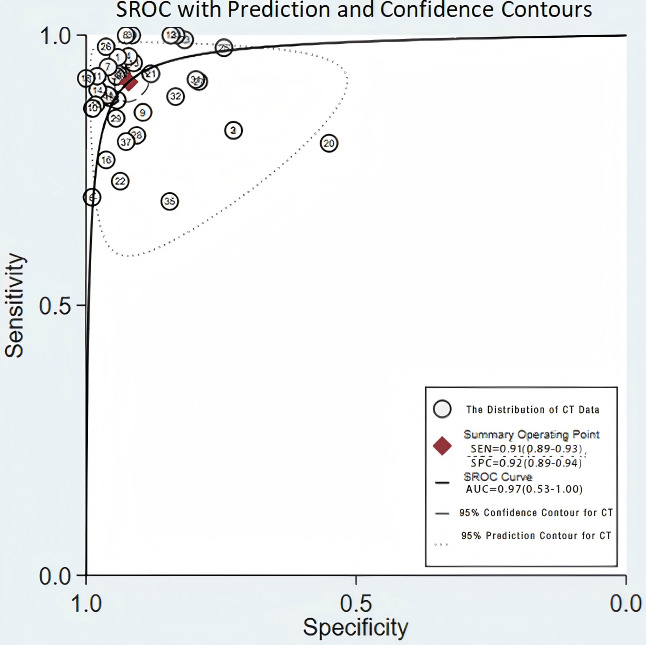
95% Confidence contour for CT.AUC:Area under the curve;CT:Computed tomography;SEN: Sensitivity; SPC: Specificity;SROC: Summary receiver operating characteristic.

**Figure 9.**
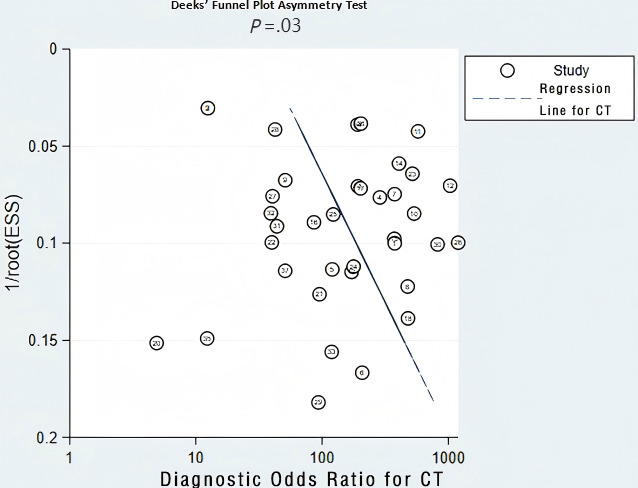
Diagnostic odds ratio for CT .CT: Computed tomography;ESS:Effective sample size.

**Figure 10.**
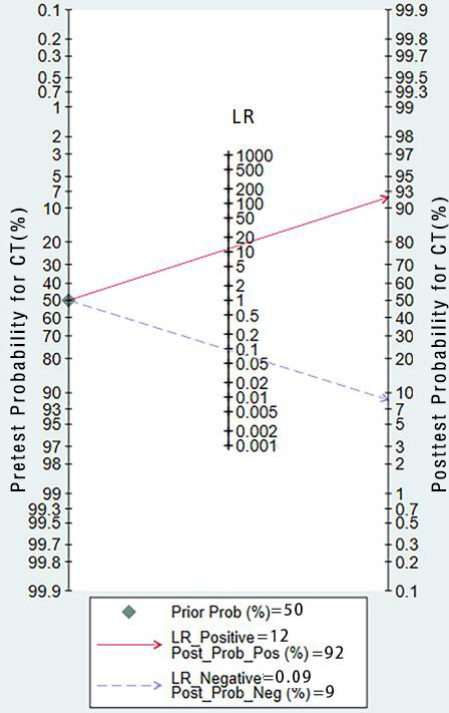
Pretest probability for CT. CT:Computed tomography;LR:Likelihood ratio.

#### Subgroup Analysis: Types of ML

##### Deep Learning

In the validation set, there were 15 diagnostic 4-fold tables for validating the CT-based DL models for diagnosing OP. The bivariate mixed-effects model was used. The pooled SEN, SPC, PLR, NLR, DOR, and SROC curve were 0.91 (95% CI 0.88‐0.94), 0.94 (95% CI 0.92‐0.96), 16.3 (95% CI 11.9‐22.3), 0.09 (95% CI 0.06‐0.13), 178 (95% CI 106‐299), and 0.98 (95% CI 0.96‐0.99), respectively (Figures S29 and S30 in Multimedia Appendix 4). Deeks’ funnel plot exhibited no marked publication bias (Figure S31 in Multimedia Appendix 4). Among the research participants included, the proportion of individuals with OP was approximately 32% (n=3197). Therefore, assuming this as the prior probability, if the ML models predicted OP, the actual probability of OP was .88. If the ML models predicted no OP, the actual probability of non-OP was .96 (Figure S32 in Multimedia Appendix 4).

##### Traditional ML

In the validation set, there were 15 diagnostic 4-fold tables for validating traditional ML models based on CT for diagnosing OP. The bivariate mixed-effects model was used. The pooled SEN, SPC, PLR, NLR, DOR, and SROC curve were 0.92 (95% CI 0.88‐0.95), 0.85 (95% CI 0.77‐0.90), 6.1 (95% CI 4.0‐9.4), 0.09 (95% CI 0.06‐0.15), 67 (95% CI 35‐128), and 0.95 (95% CI 0.93‐0.97), respectively (Figures S33 and S34 in Multimedia Appendix 4). Deeks’ funnel plot did not show notable publication bias (Figure S35 in Multimedia Appendix 4). Among the research participants, the proportion of individuals with OP was approximately 60% (n=6486). Therefore, assuming this as the prior probability, if the ML models predicted OP, the actual probability of OP was .90. If the ML models predicted no OP, the actual probability of non-OP was .88 (Figure S36 in Multimedia Appendix 4).

### Validation Set Generation Method

#### External Validation

In the OP diagnosis models constructed based on CT, validation sets for 5 diagnostic 4-fold tables were generated through external validation. The bivariate mixed-effects model was leveraged to pool data. The pooled SEN, SPC, PLR, NLR, DOR, and SROC curve were 0.88 (95% CI 0.85‐0.91), 0.97 (95% CI 0.96‐0.98), 28.4 (95% CI 20.4‐39.7), 0.12 (95% CI 0.10‐0.16), 229 (95% CI 148‐355), and 0.98 (95% CI 0.96‐0.99), respectively (Figures S37 and S38 in Multimedia Appendix 4). Deeks’ funnel plot indicated no discernible publication bias (Figure S39 in Multimedia Appendix 4). Among the included study participants, approximately 31% (n=1590) had OP. Assuming this as the prior probability, if ML predicted OP, the actual probability of OP was .93. Conversely, if ML predicted non-OP, the actual probability of non-OP was .95 (Figure S40 in Multimedia Appendix 4).

#### Random Sampling

Validation sets for 24 diagnostic 4-fold tables were generated using the random sampling method. The bivariate mixed-effects model was leveraged. The pooled SEN, SPC, PLR, NLR, DOR, and SROC were 0.91 (95% CI 0.87‐0.94), 0.90 (95% CI 0.85‐0.94), 9.4 (95% CI 6.0‐14.9), 0.10 (95% CI 0.07‐0.14), 96 (95% CI 57‐161), and 0.96 (95% CI 0.94 ‐0.97), respectively (Figures S41 and S42 in Multimedia Appendix 4). Deeks’ funnel plot presented no significant publication bias (Figure S43 in Multimedia Appendix 4). Among the included study participants, approximately 36% (n=4175) had OP. Given this as the prior probability, when the ML models predicted OP, the actual probability of OP was .84. On the other hand, when the ML models predicted non-OP, the actual probability of non-OP was .95 (Figure S44 in Multimedia Appendix 4).

### Examination Parts

#### Hip Joint

In the OP diagnostic models constructed based on CT, 6 diagnostic 4-fold tables focused on the hip joint. The bivariate mixed-effects model was used. The pooled SEN, SPC, PLR, NLR, DOR, and SROC were 0.87 (95% CI 0.83‐0.90), 0.92 (95% CI 0.81‐0.96), 10.4 (95% CI 4.4‐24.7), 0.14 (95% CI 0.10‐0.19), 76 (95% CI 24‐239), and 0.92 (95% CI 0.90‐0.94), respectively (Figures S45 and S46 in Multimedia Appendix 4). Deeks’ funnel plot did not show any marked publication bias (Figure S47 in Multimedia Appendix 4). Among the included study participants, approximately 69% (n=2719) had OP. Assuming this as the prior probability, if ML predicted OP, the actual probability of OP was .96. If the models predicted non-OP, the actual probability of OP was .77 (Figure S48 in Multimedia Appendix 4).

#### Thoracic Vertebrae

In total, 9 diagnostic 4-fold tables focused on the thoracic vertebrae. The bivariate mixed-effects model was leveraged to pool data. The pooled SEN, SPC, PLR, NLR, DOR, and SROC were 0.91 (95% CI 0.86‐0.94), 0.94 (95% CI 0.92‐0.95), 14.4 (95% CI 10.7‐19.3), 0.10 (95% CI 0.06‐0.15), 150 (95% CI 75‐300), and 0.97 (95% CI 0.95‐0.98), respectively (Figures S49 and S50 in Multimedia Appendix 4). Significant publication bias was not observed in Deeks’ funnel plot (Figure S51 in Multimedia Appendix 4). Among the encompassed study participants, approximately 29% (n=2523) had OP. Assuming this as the prior probability, if ML predicted OP, the actual probability of OP was .85. If ML predicted non-OP, the actual probability of non-OP was .96 (Figure S52 in Multimedia Appendix 4).

#### Lumbar Vertebrae

For OP diagnostic models using the lumbar vertebrae as the target part, 26 diagnostic 4-fold tables were analyzed. The bivariate mixed-effects model yielded a SEN of 0.91 (95% CI 0.87‐0.94), SPC of 0.92 (95% CI 0.86‐0.95), PLR of 10.7 (95% CI 6.7‐17.2), NLR of 0.10 (95% CI 0.07‐0.14), DOR of 110 (95% CI 63‐191), and SROC curve of 0.96 (95% CI 0.94‐0.98; Figures S53 and S54 in Multimedia Appendix 4). Deeks’ funnel plot did not reflect discernible publication bias (Figure S55 in Multimedia Appendix 4). Among the encompassed study participants, approximately 42% (n=7327) had OP. Assuming this as the prior probability, if ML predicted OP, the probability of actual OP was .89. Conversely, if ML predicted non-OP, the actual likelihood of non-OP was .93 (Figure S56 in Multimedia Appendix 4).

### ML Based on MRI

Only 3 studies have constructed diagnostic models for OP based on MRI [45,64,66], all of which used the lumbar vertebrae as the examination part. Due to the limited number of studies of this type and the substantial heterogeneity noted in the meta-analysis, the conclusions drawn lack sufficient reference significance. Therefore, this study presents only a narrative analysis of this part.

Among these, 2 studies used traditional ML models, while 1 used a DL model. The SEN of these models was 0.857, 0.872, and 0.892, and the SPC was 0.944, 0.688, and 0.892, respectively. The validation strategies used in these studies included external validation, K-fold cross-validation, and random sampling.

## Discussion

### Main Findings of This Study

Medical imaging is an indispensable tool in the diagnosis, treatment, and management of OP. Conventional imaging methods such as x-ray, CT, and MRI are pivotal clinically.

X-ray imaging enables clinicians to visually assess reductions in vertebral height, cortical bone thickness, and morphological changes in appendicular and mandibular bones, thus screening OP. However, as DXA is updated and improved, clinicians can more accurately know bone mineral density and structural parameters of the lumbar vertebrae and hip, thereby facilitating the diagnosis of OP. The World Health Organization has designated DXA as the gold standard for determining bone mineral density and diagnosing postmenopausal OP [74,75]. However, in low-resource environments and economically underdeveloped regions, the clinical application of DXA is limited due to factors such as insufficient medical knowledge and constrained health care infrastructure. In contrast, AI tools have the potential to maximize the extraction of clinically relevant information from various medical images, thereby enabling the early identification of the population with OP or low bone mass. This significantly supports the early prevention, diagnosis, and management of the disease.

### Advantages of Different Imaging Modalities in the Diagnosis of OP

This shift has diminished the application of x-rays in quantitative analysis for OP. Nevertheless, ML and DL models have improved the diagnostic performance of x-ray imaging, providing significant impetus for its broader clinical application. CT, with its high resolution, enables clinicians to observe cortical and trabecular bone integrity, offering distinct advantages in evaluating spinal OP and changes in trabecular bone volume ratios in the hip [76]. Conventional CT generates images by measuring differences in the linear attenuation coefficients of x-ray beams as they pass through various biological tissues. However, when tissues possess similar densities, such as calcium and bone, conventional CT often yields comparable Hounsfield unit values due to the use of a single x-ray energy spectrum, limiting its ability to differentiate between such tissues. In contrast, spectral CT imaging, which is based on tissue-specific photoelectric effect weighting, offers enhanced resolution in distinguishing fine bone microarchitecture. This technological advancement holds significant potential for improving the diagnostic accuracy of OP. MRI is highly efficient in assessing bone microarchitecture [77]. However, MRI is not the first choice to detect OP because of its high cost, extended scan times, and obstacles faced by patients with metallic implants or claustrophobia. Our database search corroborated that most studies have focused on x-ray and CT imaging, while comparatively fewer have investigated MRI. Nevertheless, existing evidence supports the robust diagnostic performance of ML models based on imaging data. For example, the pooled SEN and SPC of ML models based on x-ray for OP diagnosis were 0.92 (95% CI 0.88‐0.94) and 0.83 (95% CI 0.76‐0.88), respectively. Similarly, ML models developed via CT achieved SEN and SPC of 0.91 (95% CI 0.89‐0.93) and 0.92 (95% CI 0.89‐0.94), respectively. These findings demonstrate the high accuracy of x-ray and CT in OP diagnosis. In addition, quantitative ultrasound is another commonly used modality for OP detection. Quantitative ultrasound relies on 2 primary parameters: speed of sound and broadband ultrasound attenuation, which assess the ability of ultrasound waves to propagate through bone both horizontally and longitudinally [78]. In summary, diverse imaging modalities and bone types provide flexible and enriched diagnostic options for OP. Furthermore, this variety brings ample opportunities for the development of advanced ML models tailored to different imaging techniques.

### Status Quo of Research on ML

With advances in computer science, numerous researchers have sought to use these techniques in the prevention and treatment of OP. Compared with clinicians, who visually observe positive imaging features, AI-assisted tools significantly improve the efficiency and accuracy of diagnosing OP [46,60]. In addition, Yang et al [79] developed an ML-based predictive model using data from surveys on risk factors for OP, which is highly prospective for early screening and treating OP in the Hong Kong population. Similarly, ML models based on community health examinations and serum bone turnover markers have demonstrated a high area under the receiver operating characteristic curve, *F*_1_-scores, and accuracy [80,81]. These findings highlight the efficiency of ML in the diagnosis and management of OP.

### Mechanism of Image-Based ML

Image-based ML can broadly be categorized into traditional ML and DL. Traditional ML involves dividing data into a training set for model development and a test set for model validation. Through processes such as image segmentation, texture extraction, and feature selection, traditional ML models are constructed for predicting outcome events. However, the process of texture feature extraction and selection carries a significant risk of data loss. In contrast, DL incorporates feature extraction directly into the training process, thereby maximizing the retention of meaningful information within the image data. Convolutional neural networks, as a representative DL approach, can simultaneously extract and select features across multiple hidden layers to accomplish classification tasks. Moreover, DL-based models can correct image blurring in panoramic x-rays caused by patient mispositioning and mitigate the impact of metal artifacts in CT images on feature extraction [82-84]. This study further demonstrates that ML models based on x-ray and CT outperform traditional ML models, suggesting that DL is more accurate than traditional ML approaches. Image analysis using DL can leverage AI to develop more efficient and user-friendly image interpretation tools, providing valuable insights into the development of medical imaging software.

### The Impact of Validation Set Generation Methods on ML Performance

Validation methods are critical metrics for assessing the performance of ML models. These methods can be categorized into external validation and internal validation. Internal validation can be further subdivided into random sampling, leave-one-out validation, and K-fold cross-validation. External validation, which can accurately reflect the clinical applicability of ML, is widely preferred by researchers. In contrast, internal validation typically generates validation sets via random methods, which inherently carries a risk of similarity in features and distribution trends between the validation and training sets. This issue is prominent in image-based studies, where the application of ML is restricted in medical research due to the high similarity in images and parameters between internal validation sets. Although external validation offers a superior means of assessing model performance, conducting such validation requires access to independent research cohorts and often entails consideration of factors such as periods, geographical regions, populations, and health care institutions. These requirements inevitably lead to substantial increases in both the time and financial costs of research. This perspective provides an objective explanation for the limited external validation in this study.

### Advantages and Limitations

This study is the first to summarize the evidence of the application of ML based on various imaging modalities in the diagnosis of OP. This study provides theoretical support for the subsequent development of clinical scoring systems and medical software. However, our research has the following limitations: first, despite a substantial number of included studies, only a small number of studies on MRI were encompassed in view of the practicability in clinical work. Therefore, in future research, our emphasis will be put on meta-analyses involving MRI studies, aiming to evaluate the utility of ML in the diagnosis of OP through medical imaging. As a result, only a narrative review was performed, without a direct evaluation of its diagnostic performance. Most of the included studies rely primarily on internal validation, with insufficient external validation, which imposes certain limitations on the interpretability and generalizability of our findings. This study encompassed only English publications, with the majority of research originating from countries where AI is more widely applied. In addition, the external validation conducted in this study was limited, constituting an objective constraint that may have influenced the outcomes of the meta-analysis. Future studies will endeavor to comprehensively incorporate globally available literature to enhance the authority and generalizability of the conclusions.

### Conclusions

Image-based ML, particularly DL based on x-ray and CT images, is highly accurate in the diagnosis of OP. Future focus should be placed on developing AI-based software to expand its clinical applicability and enhance diagnostic precision.

## Supplementary material

10.2196/75965 Multimedia Appendix 1 Literature search strategy.

10.2196/75965 Multimedia Appendix 2 Supplementary data sheet.

10.2196/75965 Multimedia Appendix 3 Quality Assessment of Diagnostic Accuracy Studies assessment process for included studies.

10.2196/75965 Multimedia Appendix 4 Supplementary materials.

10.2196/75965 Checklist 1 PRISMA checklist.

## Abbreviations

AI: artificial intelligence
CAD: computer-aided diagnosis
CT: computed tomography
DL: deep learning
DOR: diagnostic odds ratio
DXA: dual-energy x-ray absorptiometry
ML: machine learning
MRI: magnetic resonance imaging
NLR: negative likelihood ratio
OP: osteoporosis
PLR: positive likelihood ratio
PRISMA: Preferred Reporting Items for Systematic Reviews and Meta-Analyses
SEN: sensitivity
SPC: specificity
SROC: summary receiver operating characteristic
