# Supplementary material for: The Diagnostic Value of Image-Based Machine Learning for Osteoporosis: Systematic Review and Meta-Analysis
Source: J Med Internet Res. 2026 Jan 16;28:e75965. doi: 10.2196/75965 (PMC12810749; doi:10.2196/75965)
Supplement: Multimedia Appendix 1 [file jmir-v28-e75965-s001.docx]

1. **Pubmed**

**Date Run: 2024/5/15**

| Search number | Query | Results |
| --- | --- | --- |
| #1 | "Osteoporosis"[Mesh] | 64652 |
| #2 | ((((((((((((((((Osteoporosis[Title/Abstract]) OR (Osteoporosis[Title/Abstract])) OR (Osteoporoses[Title/Abstract])) OR (Post-Traumatic Osteoporoses[Title/Abstract])) OR (Post-Traumatic Osteoporosis[Title/Abstract])) OR (Senile Osteoporoses[Title/Abstract])) OR (Senile Osteoporosis[Title/Abstract])) OR (Age-Related Bone Loss[Title/Abstract])) OR (Age-Related Bone Losses[Title/Abstract])) OR (Age-Related Osteoporosis[Title/Abstract])) OR (Age Related Osteoporosis[Title/Abstract])) ) OR (Age-Related Osteoporoses[Title/Abstract])) OR (Perimenopausal Bone Loss[Title/Abstract])) OR (Postmenopausal Bone Losses[Title/Abstract])) OR (osteoporotic decalcification[Title/Abstract])) OR (pathologic decalcification[Title/Abstract]) | 88318 |
| #3 | (((((((((((((((((Osteoporosis[Title/Abstract]) OR (Osteoporosis[Title/Abstract])) OR (Osteoporoses[Title/Abstract])) OR (Post-Traumatic Osteoporoses[Title/Abstract])) OR (Post-Traumatic Osteoporosis[Title/Abstract])) OR (Senile Osteoporoses[Title/Abstract])) OR (Senile Osteoporosis[Title/Abstract])) OR (Age-Related Bone Loss[Title/Abstract])) OR (Age-Related Bone Losses[Title/Abstract])) OR (Age-Related Osteoporosis[Title/Abstract])) OR (Age Related Osteoporosis[Title/Abstract])) ) OR (Age-Related Osteoporoses[Title/Abstract])) OR (Perimenopausal Bone Loss[Title/Abstract])) OR (Postmenopausal Bone Losses[Title/Abstract])) OR (osteoporotic decalcification[Title/Abstract])) OR (pathologic decalcification[Title/Abstract])) OR ("Osteoporosis"[Mesh]) | 106182 |
| #4 | machine learning[MeSH Terms] | 68213 |
| #5 | ((((((((((((((((((((((((((((machine learning[Title/Abstract]) OR (Transfer Learning[Title/Abstract])) OR (Deep learning[Title/Abstract])) OR (Ensemble Learning[Title/Abstract])) OR (artificial intelligence[Title/Abstract])) OR (random forest[Title/Abstract])) OR (neural network[Title/Abstract])) OR (neural networks[Title/Abstract])) OR (K-Nearest Neighbor[Title/Abstract])) OR (CNN[Title/Abstract])) OR (Support vector machine[Title/Abstract])) OR (SVM[Title/Abstract])) OR (Gradient Boosting Machine[Title/Abstract])) OR (Nomogram[Title/Abstract])) OR (XGBoost[Title/Abstract])) OR (Adaboost[Title/Abstract])) OR (Decision tree[Title/Abstract])) OR (ResNet-50[Title/Abstract])) OR (ResNet[Title/Abstract])) OR (AlexNet[Title/Abstract])) OR (VGGNet[Title/Abstract])) OR (GoogLeNet[Title/Abstract])) OR (LSTMs[Title/Abstract])) OR (VGGNet[Title/Abstract])) OR (Naive Bayesian[Title/Abstract])) OR (Multilayer perceptron[Title/Abstract])) OR (Bayesian network[Title/Abstract])) OR (Radiomics[Title/Abstract])) OR (Radiomic[Title/Abstract]) | 327479 |
| #6 | (machine learning[MeSH Terms]) OR (((((((((((((((((((((((((((((machine learning[Title/Abstract]) OR (Transfer Learning[Title/Abstract])) OR (Deep learning[Title/Abstract])) OR (Ensemble Learning[Title/Abstract])) OR (artificial intelligence[Title/Abstract])) OR (random forest[Title/Abstract])) OR (neural network[Title/Abstract])) OR (neural networks[Title/Abstract])) OR (K-Nearest Neighbor[Title/Abstract])) OR (CNN[Title/Abstract])) OR (Support vector machine[Title/Abstract])) OR (SVM[Title/Abstract])) OR (Gradient Boosting Machine[Title/Abstract])) OR (Nomogram[Title/Abstract])) OR (XGBoost[Title/Abstract])) OR (Adaboost[Title/Abstract])) OR (Decision tree[Title/Abstract])) OR (ResNet-50[Title/Abstract])) OR (ResNet[Title/Abstract])) OR (AlexNet[Title/Abstract])) OR (VGGNet[Title/Abstract])) OR (GoogLeNet[Title/Abstract])) OR (LSTMs[Title/Abstract])) OR (VGGNet[Title/Abstract])) OR (Naive Bayesian[Title/Abstract])) OR (Multilayer perceptron[Title/Abstract])) OR (Bayesian network[Title/Abstract])) OR (Radiomics[Title/Abstract])) OR (Radiomic[Title/Abstract])) | 332822 |
| #7 | ((((((((((((((((((Osteoporosis[Title/Abstract]) OR (Osteoporosis[Title/Abstract])) OR (Osteoporoses[Title/Abstract])) OR (Post-Traumatic Osteoporoses[Title/Abstract])) OR (Post-Traumatic Osteoporosis[Title/Abstract])) OR (Senile Osteoporoses[Title/Abstract])) OR (Senile Osteoporosis[Title/Abstract])) OR (Age-Related Bone Loss[Title/Abstract])) OR (Age-Related Bone Losses[Title/Abstract])) OR (Age-Related Osteoporosis[Title/Abstract])) OR (Age Related Osteoporosis[Title/Abstract])) ) OR (Age-Related Osteoporoses[Title/Abstract])) OR (Perimenopausal Bone Loss[Title/Abstract])) OR (Postmenopausal Bone Losses[Title/Abstract])) OR (osteoporotic decalcification[Title/Abstract])) OR (pathologic decalcification[Title/Abstract])) OR ("Osteoporosis"[Mesh])) AND ((machine learning[MeSH Terms]) OR (((((((((((((((((((((((((((((machine learning[Title/Abstract]) OR (Transfer Learning[Title/Abstract])) OR (Deep learning[Title/Abstract])) OR (Ensemble Learning[Title/Abstract])) OR (artificial intelligence[Title/Abstract])) OR (random forest[Title/Abstract])) OR (neural network[Title/Abstract])) OR (neural networks[Title/Abstract])) OR (K-Nearest Neighbor[Title/Abstract])) OR (CNN[Title/Abstract])) OR (Support vector machine[Title/Abstract])) OR (SVM[Title/Abstract])) OR (Gradient Boosting Machine[Title/Abstract])) OR (Nomogram[Title/Abstract])) OR (XGBoost[Title/Abstract])) OR (Adaboost[Title/Abstract])) OR (Decision tree[Title/Abstract])) OR (ResNet-50[Title/Abstract])) OR (ResNet[Title/Abstract])) OR (AlexNet[Title/Abstract])) OR (VGGNet[Title/Abstract])) OR (GoogLeNet[Title/Abstract])) OR (LSTMs[Title/Abstract])) OR (VGGNet[Title/Abstract])) OR (Naive Bayesian[Title/Abstract])) OR (Multilayer perceptron[Title/Abstract])) OR (Bayesian network[Title/Abstract])) OR (Radiomics[Title/Abstract])) OR (Radiomic[Title/Abstract]))) | 685 |

1. **Cochrane**

**Date Run: 16/05/2024 14:54:58**

| Search number | Query | Results |
| --- | --- | --- |
| #1 | (Perimenopausal Bone Loss):ti,ab,kw OR (Postmenopausal Bone Losses):ti,ab,kw OR (osteoporotic decalcification):ti,ab,kw OR (pathologic decalcification):ti,ab,kw | 100 |
| #2 | (Osteoporoses):ti,ab,kw OR (Post-Traumatic Osteoporoses):ti,ab,kw OR (Post-Traumatic Osteoporosis):ti,ab,kw OR (Senile Osteoporoses):ti,ab,kw OR (Senile Osteoporosis):ti,ab,kw | 128 |
| #3 | (Age-Related Bone Loss):ti,ab,kw OR (Age-Related Bone Losses):ti,ab,kw OR (Age-Related Osteoporosis):ti,ab,kw OR (Age Related Osteoporosis):ti,ab,kw OR (Age-Related Osteoporoses):ti,ab,kw | 859 |
| #4 | MeSH descriptor: [Osteoporosis] explode all trees | 5492 |
| #5 | #1 OR #2 OR #3 OR #4 | 6194 |
| #6 | MeSH descriptor: [Machine Learning] explode all trees | 983 |
| #7 | (Transfer Learning):ti,ab,kw OR (Deep learning):ti,ab,kw OR (Ensemble Learning):ti,ab,kw OR (artificial intelligence):ti,ab,kw OR (random forest):ti,ab,kw | 5840 |
| #8 | (neural network):ti,ab,kw OR (neural networks):ti,ab,kw OR (K-Nearest Neighbor):ti,ab,kw OR (CNN):ti,ab,kw OR (Support vector machine):ti,ab,kw | 4167 |
| #9 | (SVM):ti,ab,kw OR (Gradient Boosting Machine):ti,ab,kw OR (Nomogram):ti,ab,kw OR (XGBoost):ti,ab,kw OR (Adaboost):ti,ab,kw | 2300 |
| #10 | (Decision tree):ti,ab,kw OR (ResNet-50):ti,ab,kw OR (ResNet):ti,ab,kw OR (AlexNet):ti,ab,kw OR (VGGNet):ti,ab,kw | 1041 |
| #11 | (GoogLeNet):ti,ab,kw OR (LSTMs):ti,ab,kw OR (VGGNet):ti,ab,kw OR (Naive Bayesian):ti,ab,kw OR (Multilayer perceptron):ti,ab,kw | 185 |
| #12 | (Bayesian network):ti,ab,kw OR (Radiomics):ti,ab,kw OR (Radiomic):ti,ab,kw | 1100 |
| #13 | #6 OR #7 OR #8 OR #9 OR #10 OR #11 OR #12 | 12563 |
| #14 | #5 AND #13 | 15 |

1. **Embase**

**Date Run: 2024/5/15**

| Search number | Query | Results |
| --- | --- | --- |
| #1 | 'osteoporosis'/exp | 161805 |
| #2 | 'osteoporosis'/exp OR osteoporosis OR osteoporoses:ab,ti OR 'post-traumatic osteoporoses':ab,ti OR 'post-traumatic osteoporosis':ab,ti OR 'senile osteoporoses':ab,ti OR 'senile osteoporosis':ab,ti OR 'age-related bone loss':ab,ti OR 'age-related bone losses':ab,ti OR 'age-related osteoporosis':ab,ti OR 'age related osteoporosis':ab,ti OR 'age-related osteoporoses':ab,ti OR 'perimenopausal bone loss':ab,ti OR 'postmenopausal bone losses':ab,ti OR 'osteoporotic decalcification':ab,ti OR 'pathologic decalcification':ab,ti | 207054 |
| #3 | machine learning OR 'transfer learning':ab,ti OR 'deep learning':ab,ti OR 'ensemble learning':ab,ti OR 'artificial intelligence':ab,ti OR 'random forest':ab,ti OR 'neural network':ab,ti OR 'neural networks':ab,ti OR 'k-nearest neighbor':ab,ti OR cnn:ab,ti OR 'support vector machine':ab,ti OR svm:ab,ti OR 'gradient boosting machine':ab,ti OR nomogram:ab,ti OR xgboost:ab,ti OR adaboost:ab,ti OR 'decision tree':ab,ti OR 'resnet 50':ab,ti OR resnet:ab,ti OR alexnet:ab,ti OR googlenet:ab,ti OR lstms:ab,ti OR vggnet:ab,ti OR 'naive bayesian':ab,ti OR 'multilayer perceptron':ab,ti OR 'bayesian network':ab,ti OR radiomics:ab,ti OR radiomic:ab,ti | 463658 |
| #4 | 'machine learning'/exp | 475762 |
| #5 | #1 OR #2 | 207054 |
| #6 | #3 OR #4 | 670754 |
| #7 | #5 AND #6 | 1942 |

1. **Web of science**

**Date Run:Wed May 15 2024 17:21:37 GMT+0800 (GMT+08:00)**

| Search number | Query | Results |
| --- | --- | --- |
| #1 | Osteoporosis (Topic) OR Osteoporoses (Topic) OR Post-Traumatic Osteoporoses (Topic) OR Post-Traumatic Osteoporosis (Topic) OR Senile Osteoporoses (Topic) OR Senile Osteoporosis (Topic) OR Age-Related Bone Loss (Topic) OR Age-Related Bone Losses (Topic) OR Age-Related Osteoporosis (Topic) OR Age Related Osteoporosis (Topic) OR Age-Related Osteoporoses (Topic) OR Perimenopausal Bone Loss (Topic) OR Postmenopausal Bone Losses (Topic) OR osteoporotic decalcification (Topic) OR pathologic decalcification (Topic) | 104470 |
| #2 | machine learning (Topic) OR Transfer Learning (Topic) OR Deep learning (Topic) OR Ensemble Learning (Topic) OR artificial intelligence (Topic) OR random forest (Topic) OR neural network (Topic) OR neural networks (Topic) OR K-Nearest Neighbor (Topic) OR CNN (Topic) OR Support vector machine (Topic) OR SVM (Topic) OR Gradient Boosting Machine (Topic) OR Nomogram (Topic) OR XGBoost (Topic) OR Adaboost (Topic) OR Decision tree (Topic) OR ResNet-50 (Topic) OR ResNet (Topic) OR AlexNet (Topic) OR VGGNet (Topic) OR GoogLeNet (Topic) OR LSTMs (Topic) OR VGGNet (Topic) OR Naive Bayesian (Topic) OR Multilayer perceptron (Topic) OR Bayesian network (Topic) OR Radiomics (Topic) OR Radiomic (Topic) | 968570 |
| #3 | #1 AND #2 | 785 |
