# Supplementary material for: The Diagnostic Value of Image-Based Machine Learning for Osteoporosis: Systematic Review and Meta-Analysis
Source: J Med Internet Res. 2026 Jan 16;28:e75965. doi: 10.2196/75965 (PMC12810749; doi:10.2196/75965)
Supplement: Multimedia Appendix 4 [file jmir-v28-e75965-s004.doc]

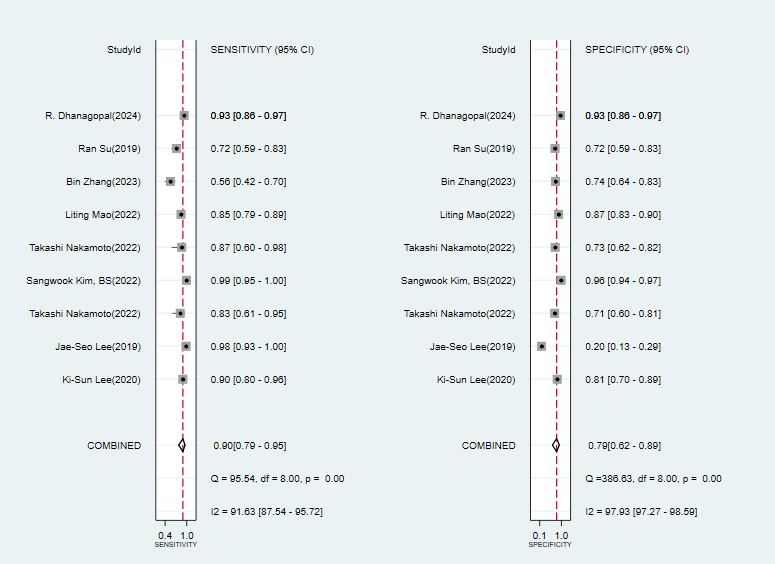


Supplementary Figure 1. Forest plot of sensitivity and specificity for the deep learning models constructed based on X-ray for diagnosing osteoporosis


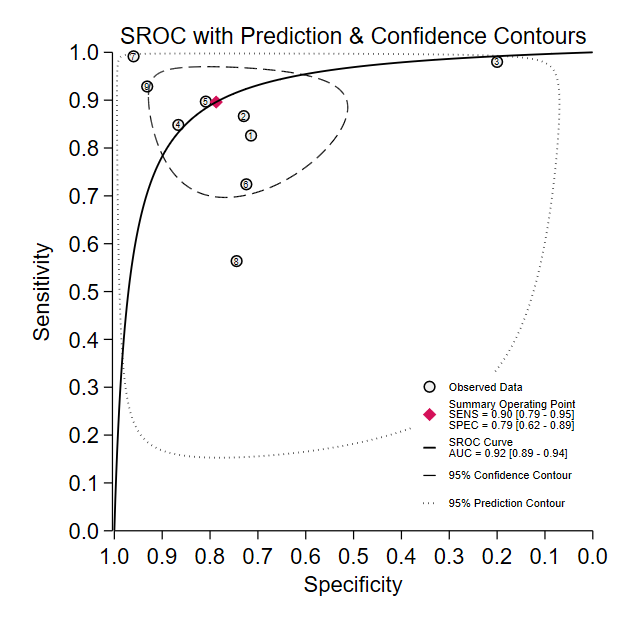


Supplementary Figure 2. SROC for the deep learning models constructed based on X-ray for diagnosing osteoporosis


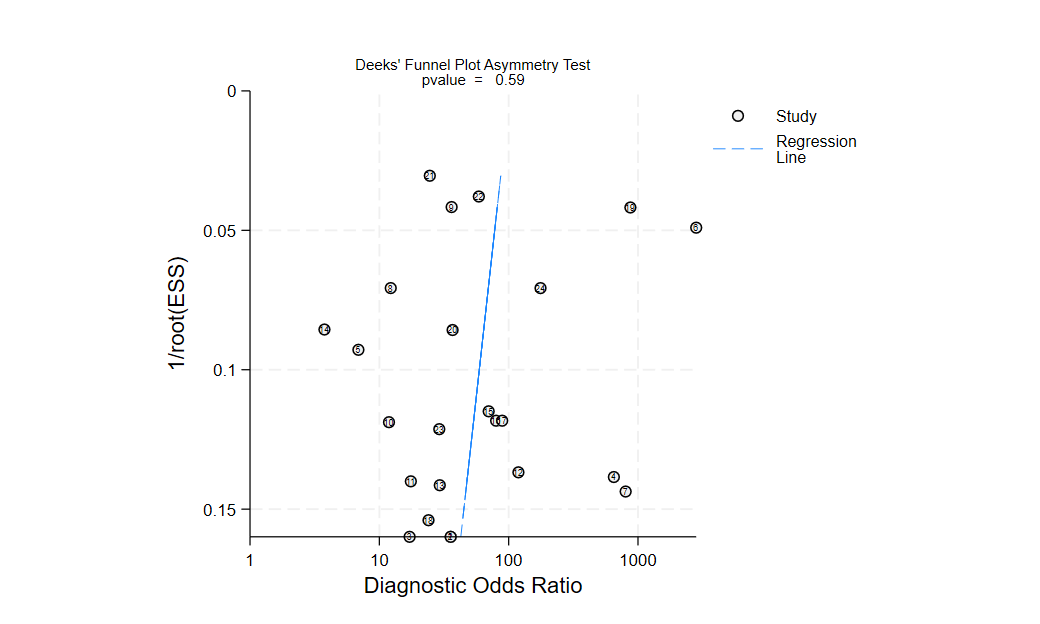


Supplementary Figure 3. Deek’s funnel plot for the deep learning models constructed based on X-ray for diagnosing osteoporosis


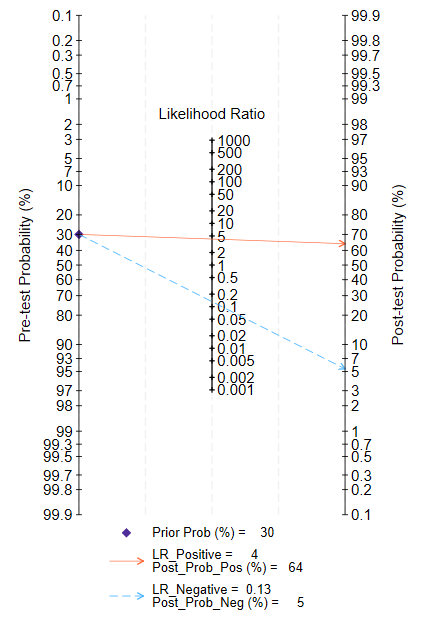


Supplementary Figure 4. Nomogram for the deep learning models constructed based on X-ray for diagnosing osteoporosis


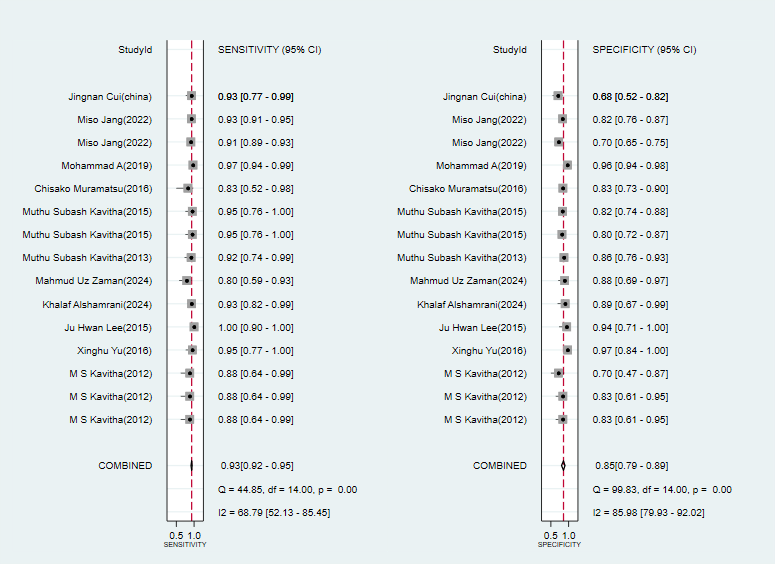


Supplementary Figure 5. Forest plot of sensitivity and specificity for the traditional machine learning models constructed based on X-ray for diagnosing osteoporosis


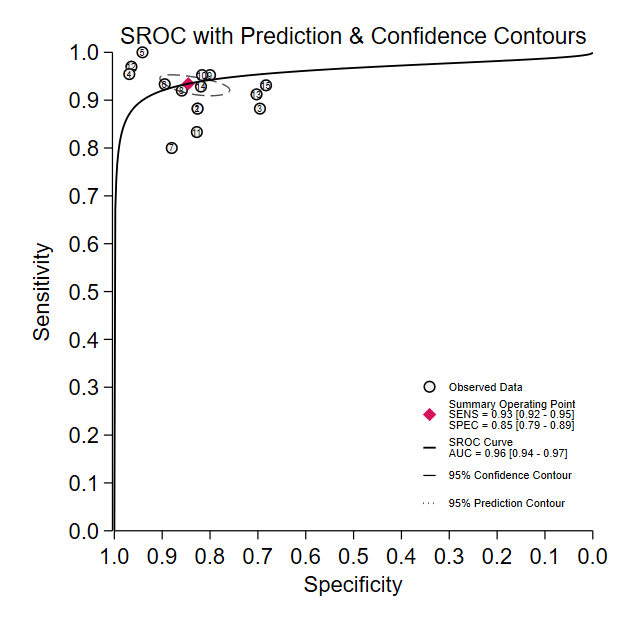


Supplementary Figure 6. SROC curve for the traditional machine learning models constructed based on X-ray for diagnosing osteoporosis


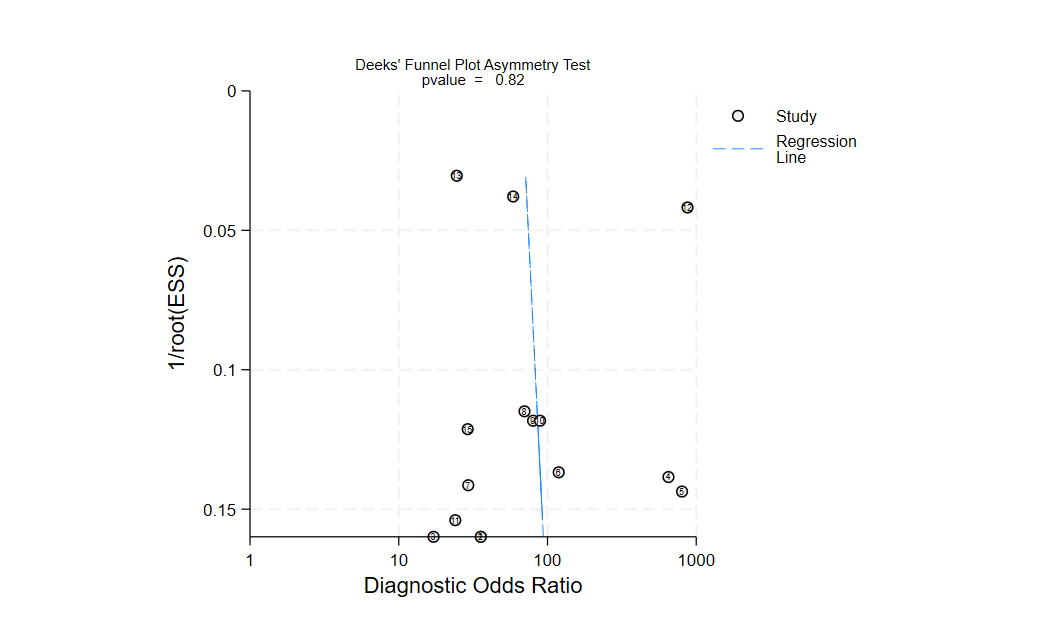


Supplementary Figure 7. Deek’s funnel plot for the traditional machine learning models constructed based on X-ray for diagnosing osteoporosis


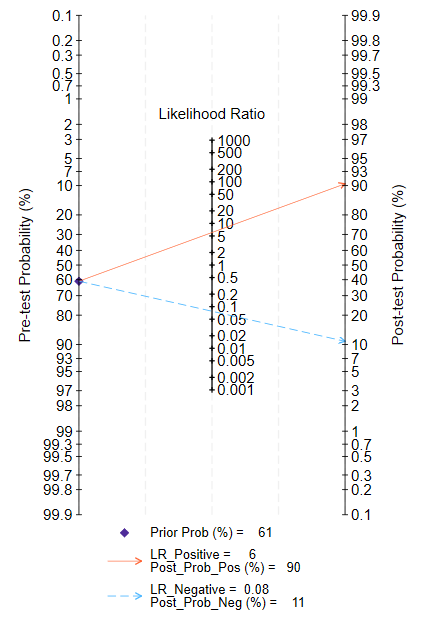


Supplementary Figure 8. Nomogram for the traditional machine learning models constructed based on X-ray for diagnosing osteoporosis


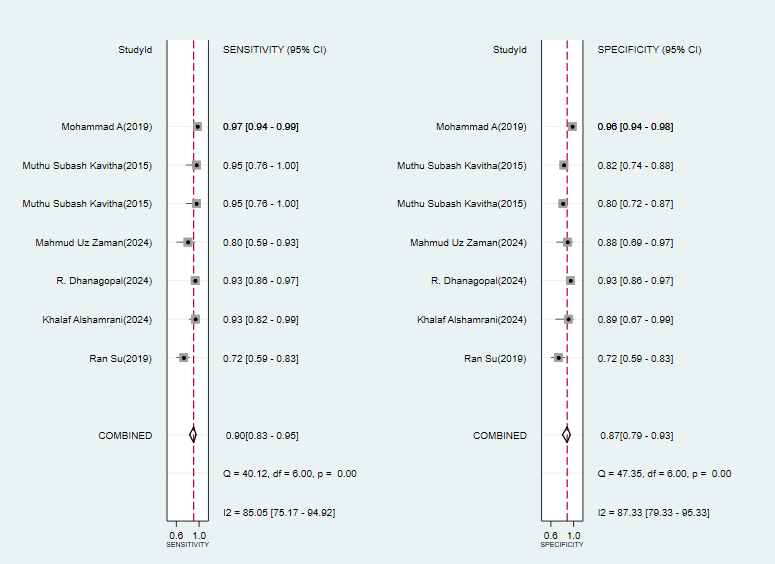


Supplementary Figure 9. Forest plot of sensitivity and specificity of X-ray-based models based on K-fold cross-validation


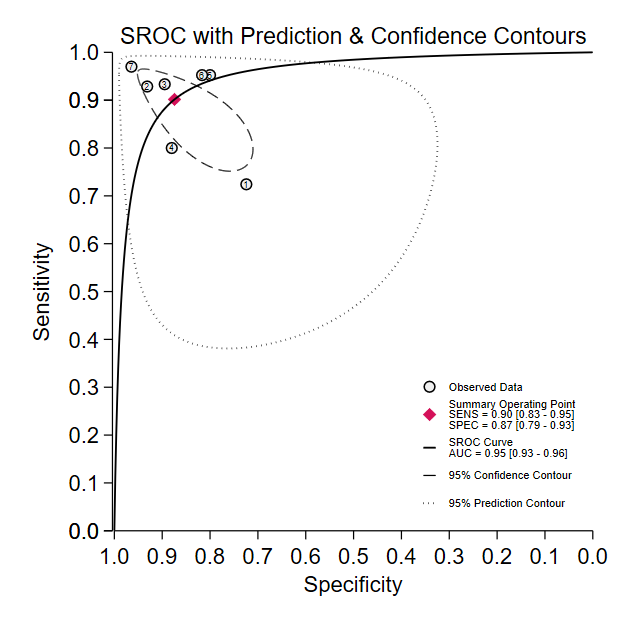


Supplementary Figure 10. SROC curve of X-ray-based models based on K-fold cross-validation


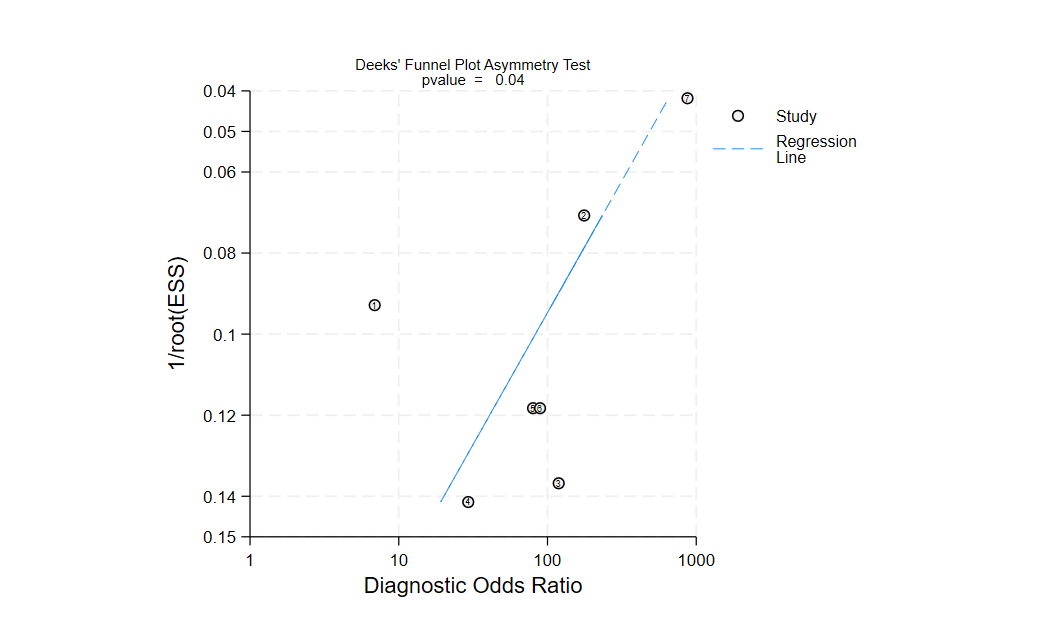


Supplementary Figure 11. Deek’s funnel plot of X-ray-based models based on K-fold cross-validation


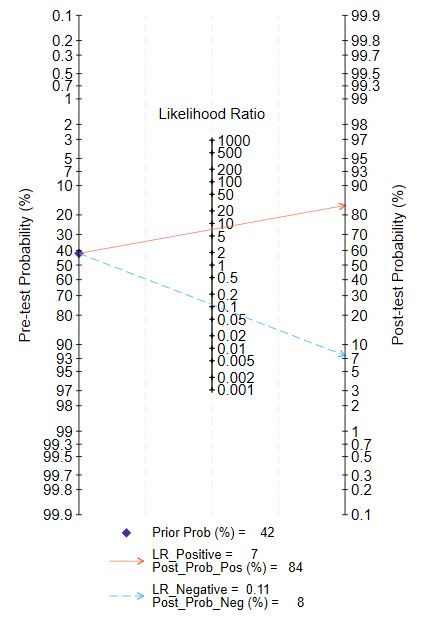


Supplementary Figure 12. Nomogram of X-ray-based models based on K-fold cross-validation


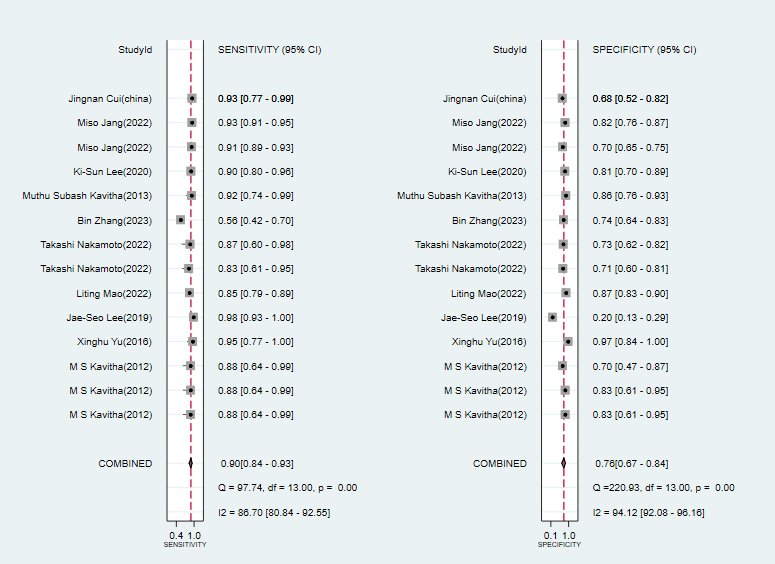


Supplementary Figure 13. Forest plot of sensitivity and specificity of X-ray-based models based on random sampling


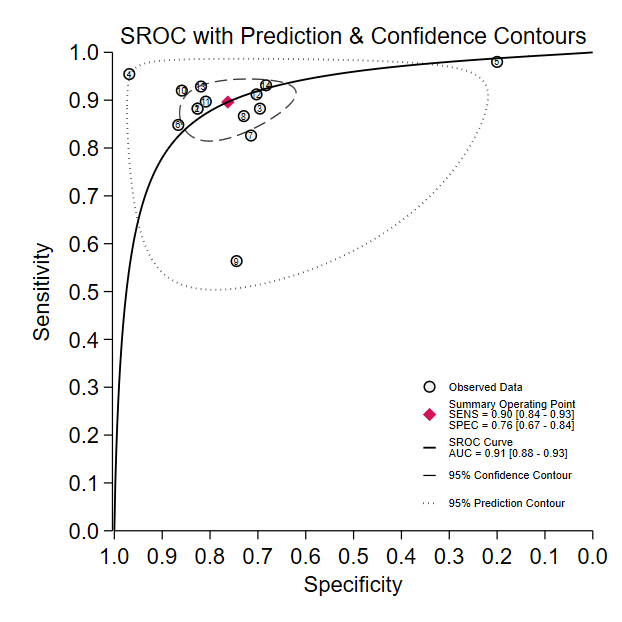


Supplementary Figure 14. SROC curve of X-ray-based models based on random sampling


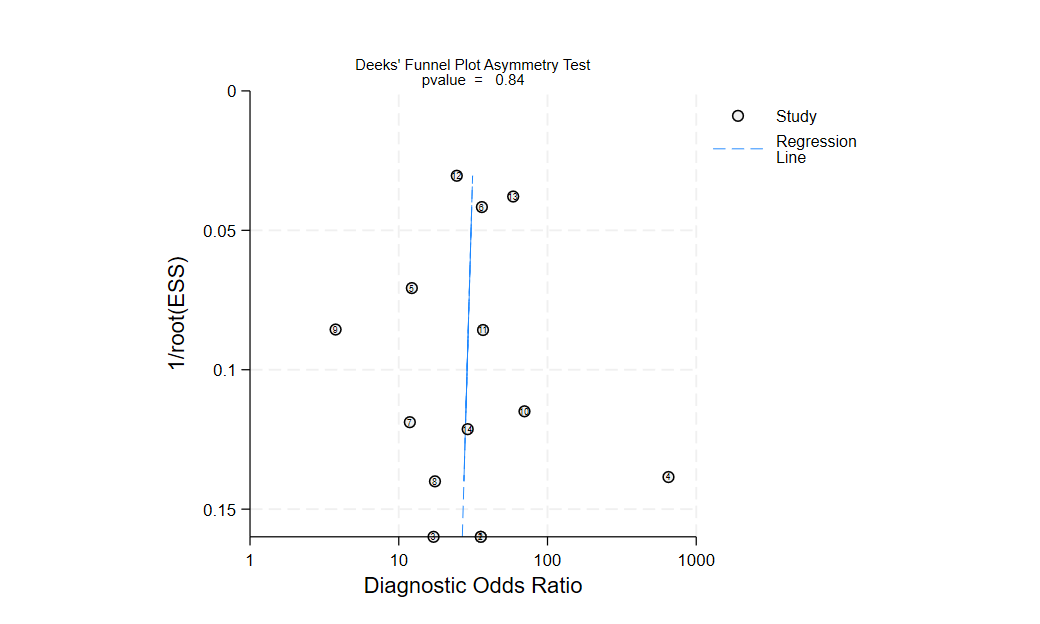


Supplementary Figure 15. Deek’s funnel plot of X-ray-based models based on random sampling


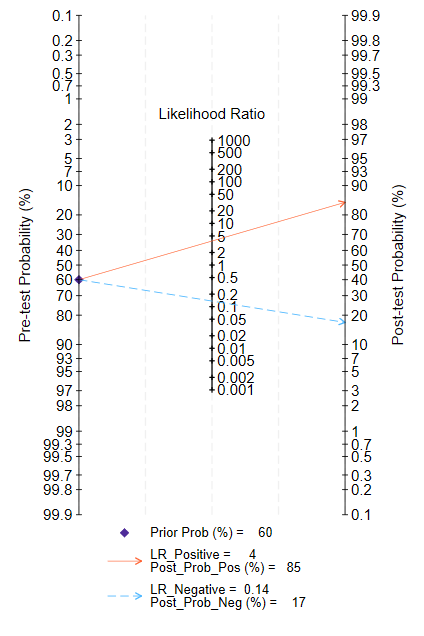


Supplementary Figure 16. Nomogram of X-ray-based models based on random sampling


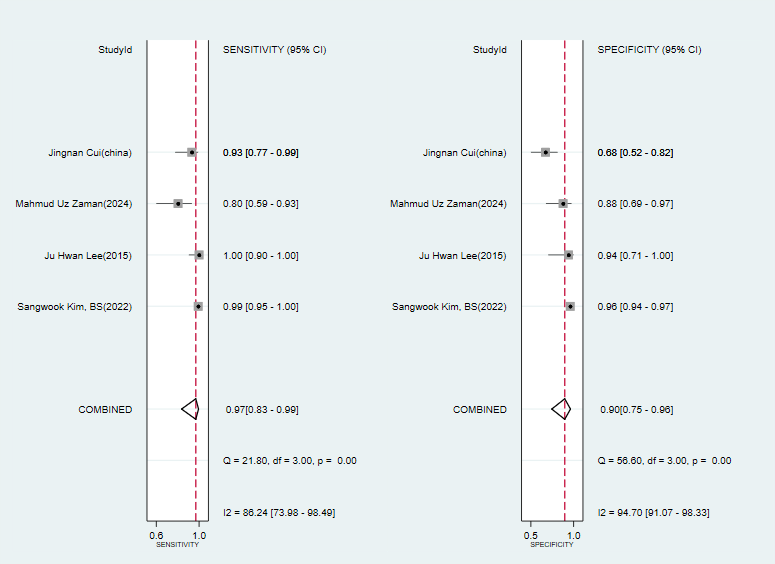


Supplementary Figure 17. Forest plot of sensitivity and specificity of X-ray-based models for limbs


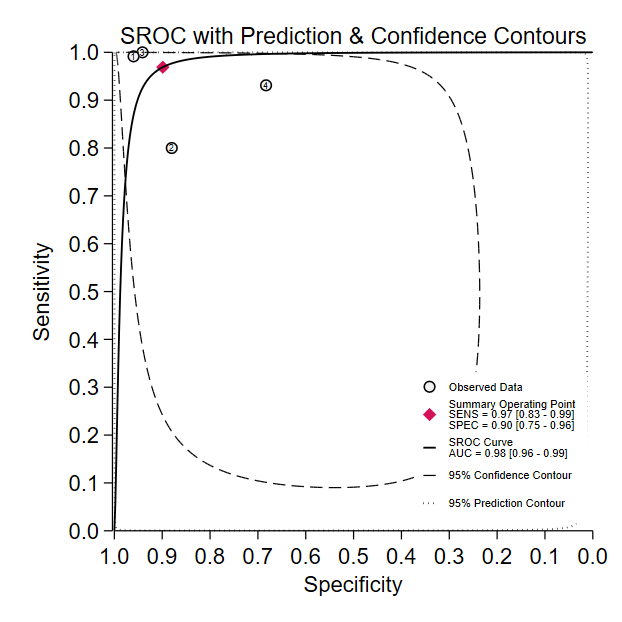


Supplementary Figure 18. SROC curve of X-ray-based models for limbs


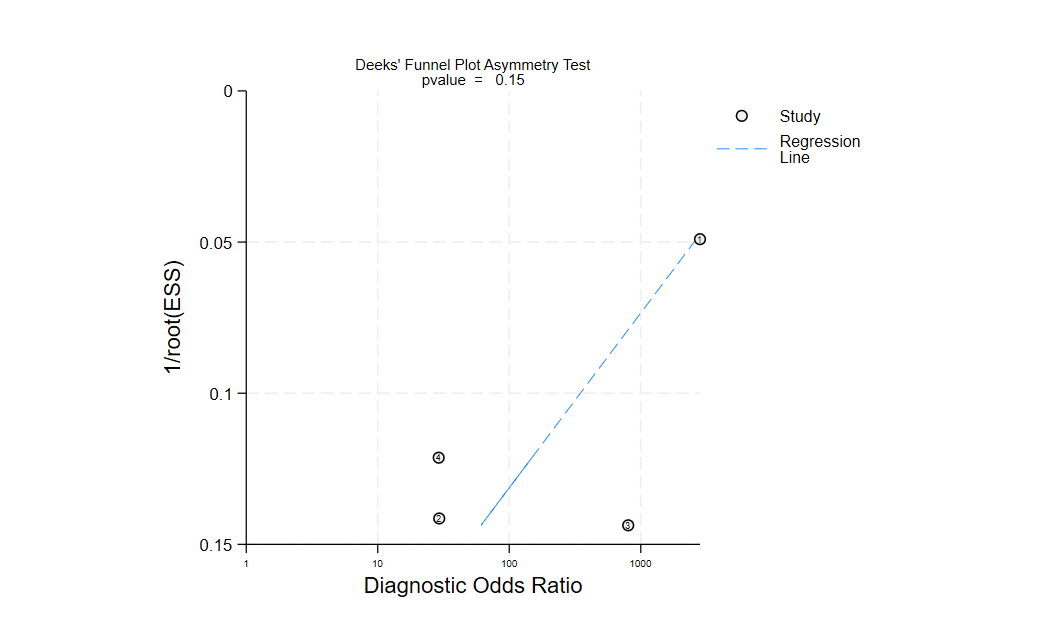


Supplementary Figure 19. Deek’s funnel plot of X-ray-based models for limbs


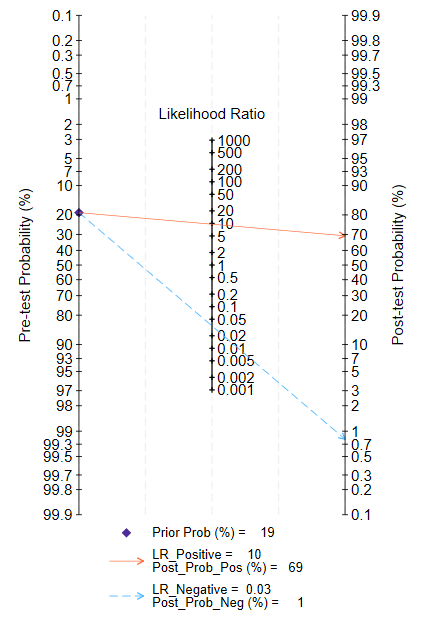


Supplementary Figure 20. Nomogram of X-ray-based models for limbs


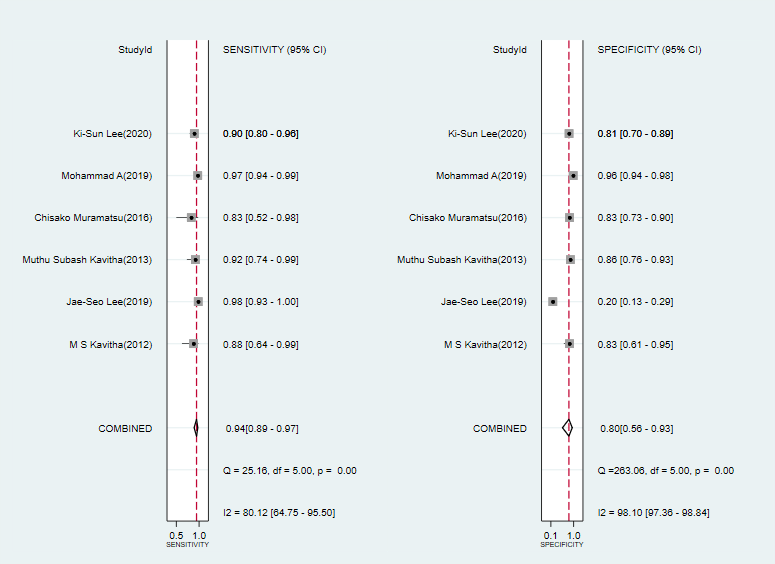


Supplementary Figure 21. Forest plot of sensitivity and specificity of X-ray-based models for mandible


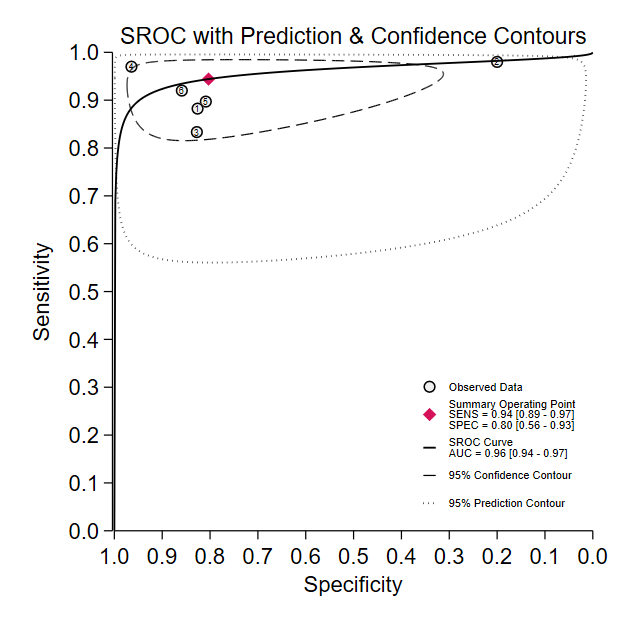


Supplementary Figure 22. SROC curve of X-ray-based models for mandible


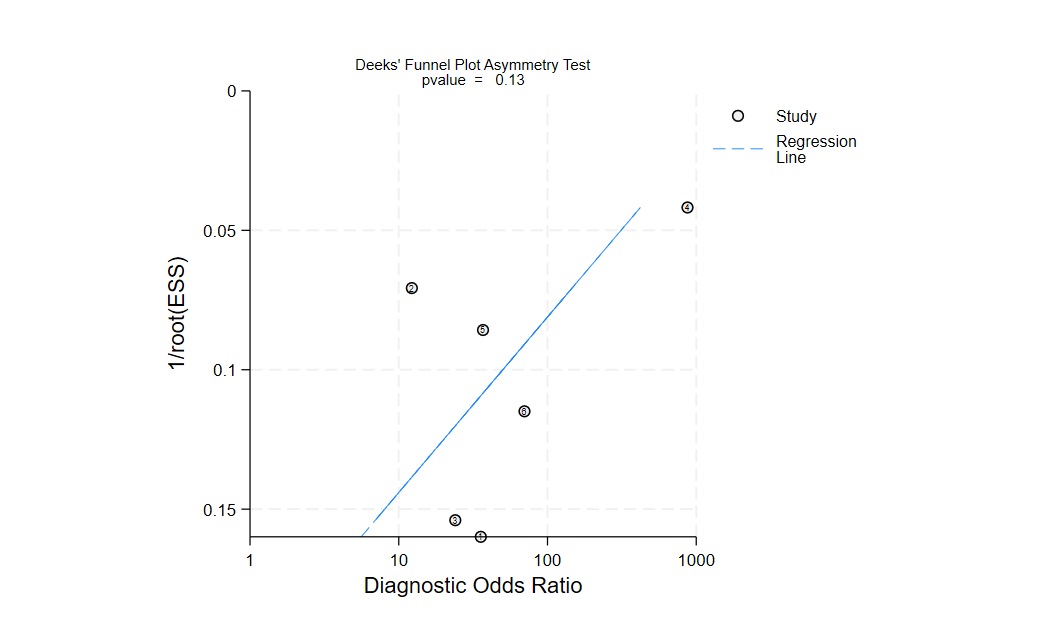


Supplementary Figure 23. Deek’s funnel plot of X-ray-based models for mandible


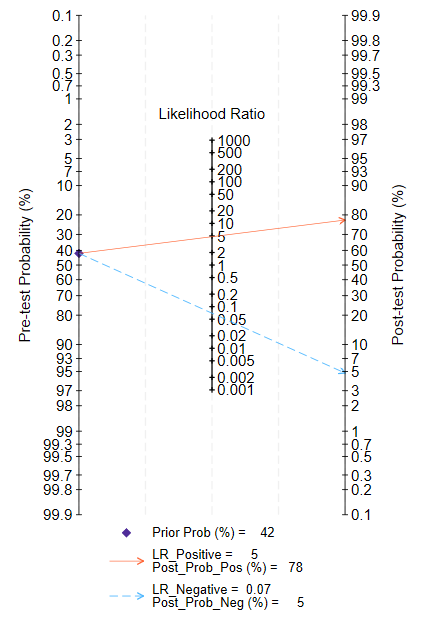


Supplementary Figure 24. Nomogram of X-ray-based models based on mandible


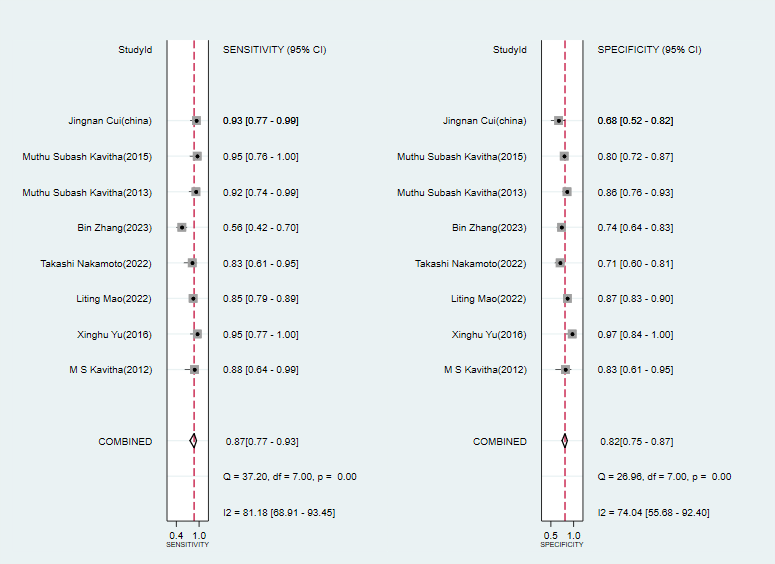


Supplementary Figure 25. Forest plot of sensitivity and specificity of X-ray-based models for lumbar vertebrae


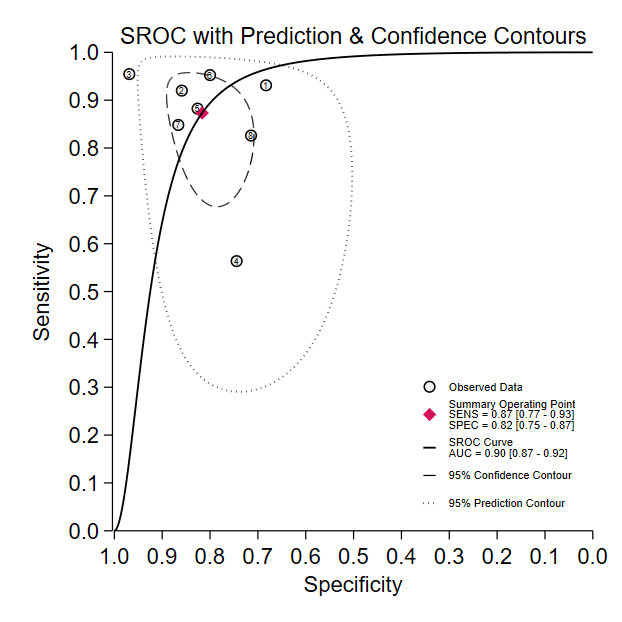


Supplementary Figure 26. SROC curve of X-ray-based models for lumbar vertebrae


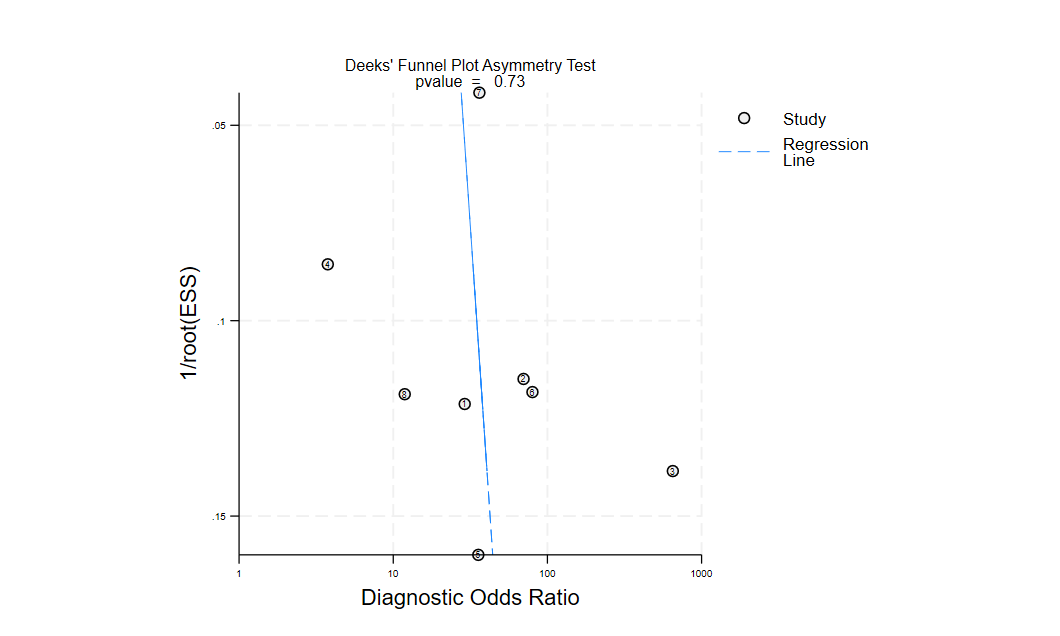


Supplementary Figure 27. Deek’s funnel plot of X-ray-based models for lumbar vertebrae


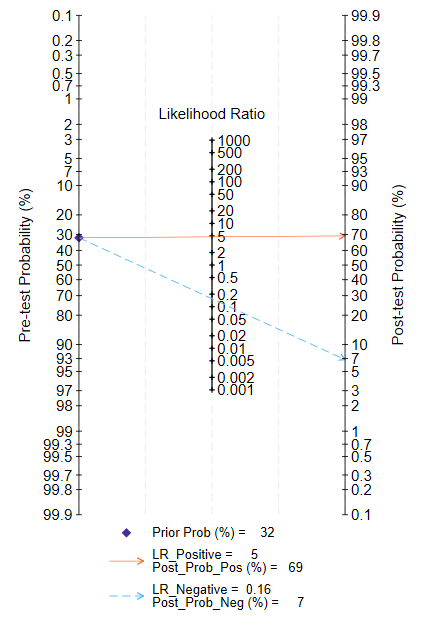


Supplementary Figure 28. Nomogram of X-ray-based models for lumbar vertebrae


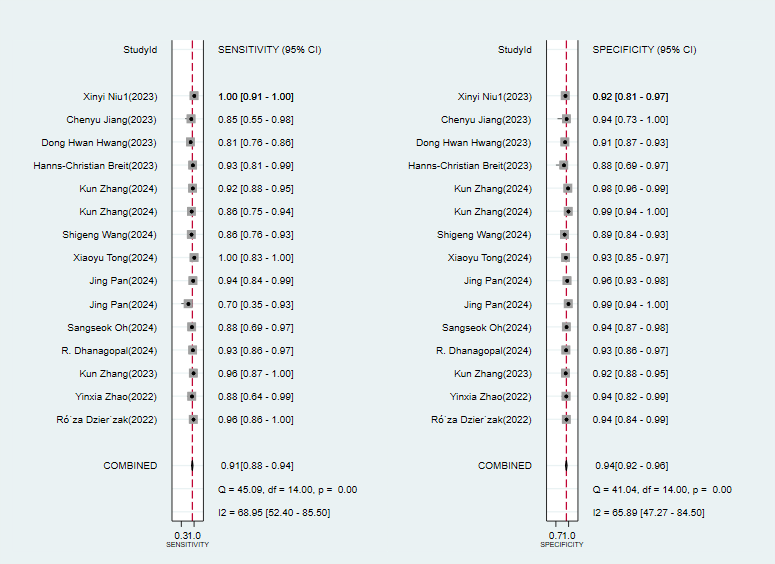


Supplementary Figure 29. Forest plot of sensitivity and specificity for the deep learning models constructed based on CT for diagnosing osteoporosis


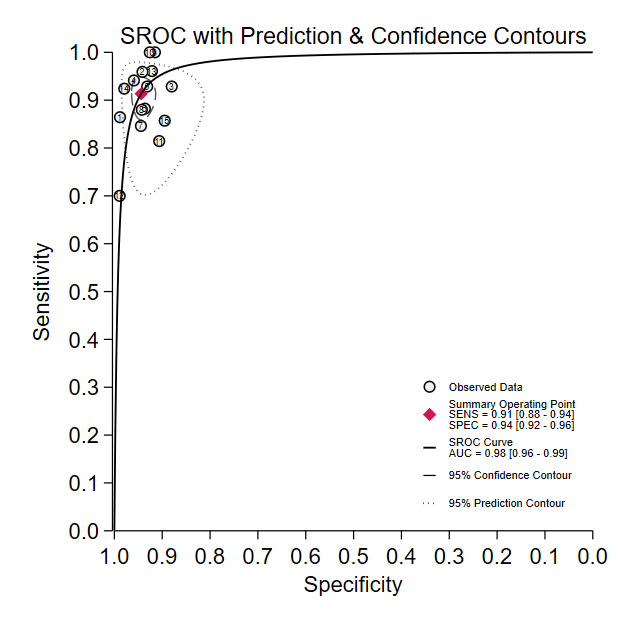


Supplementary Figure 30. SROC curve for the deep learning models constructed based on CT for diagnosing osteoporosis


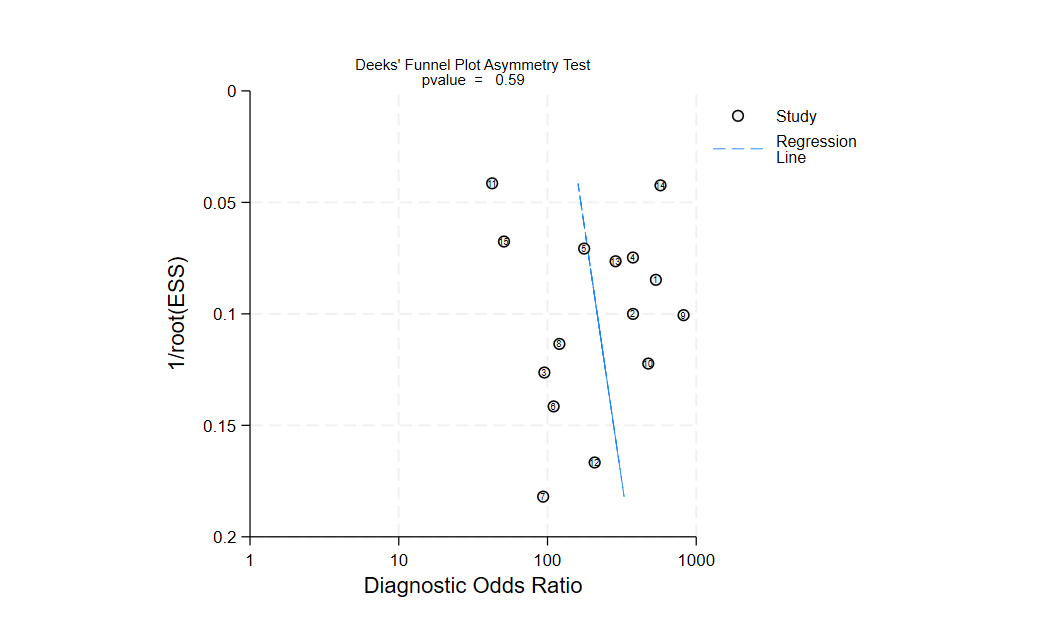


Supplementary Figure 31. Deek’s funnel plot for the deep learning models constructed based on CT for diagnosing osteoporosis


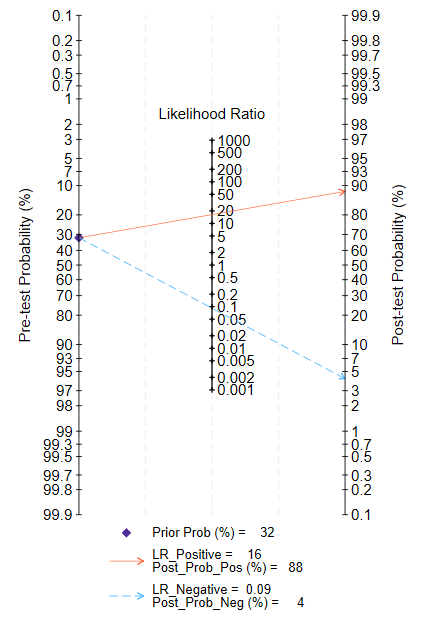


Supplementary Figure 32. Nomogram for the deep learning models constructed based on CT for diagnosing osteoporosis


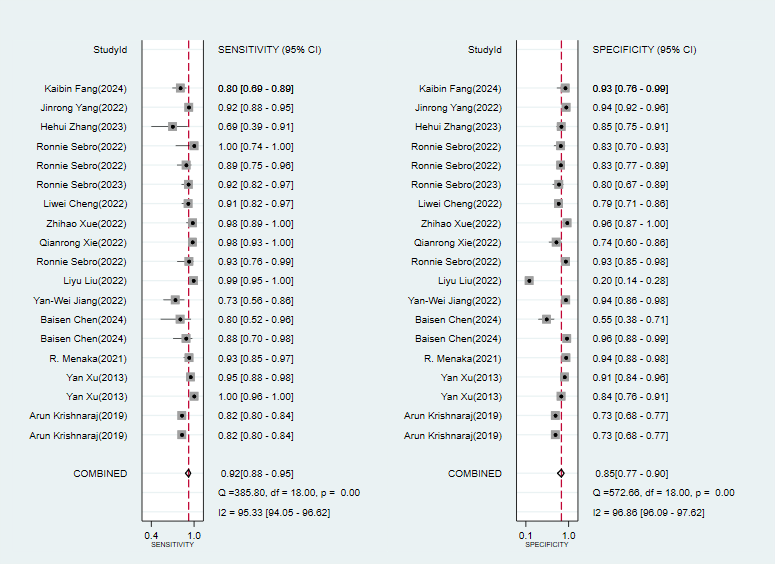


Supplementary Figure 33. Forest plot of sensitivity and specificity for the traditional machine learning models constructed based on CT for diagnosing osteoporosis


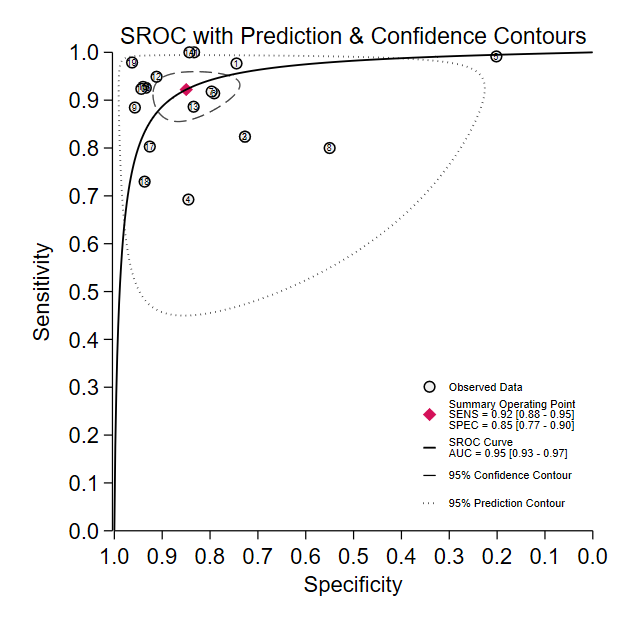


Supplementary Figure 34. SROC curve for the traditional machine learning models constructed based on CT for diagnosing osteoporosis


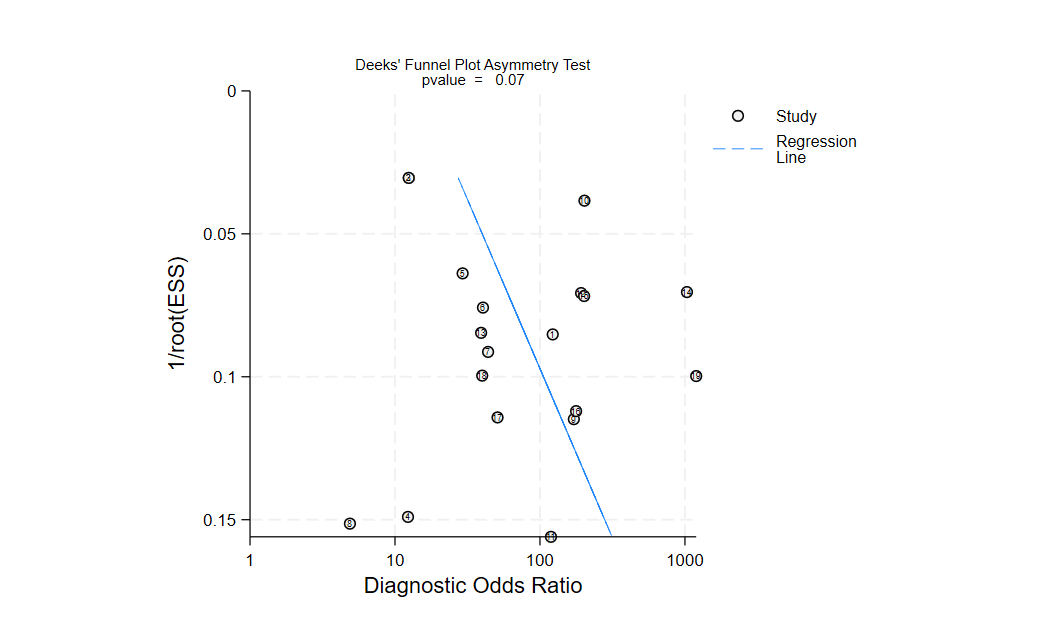


Supplementary Figure 35. Deek’s funnel plot for the traditional machine learning models constructed based on CT for diagnosing osteoporosis


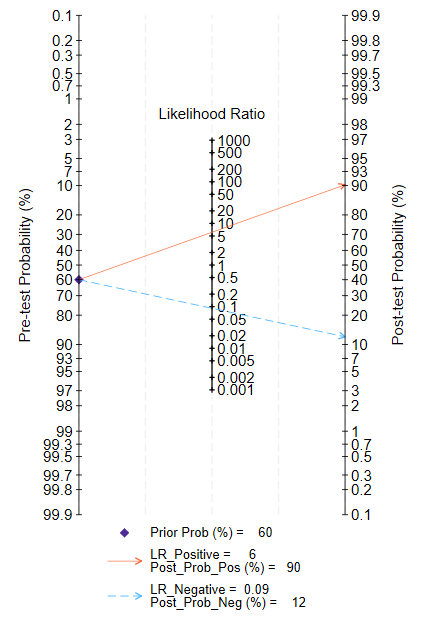


Supplementary Figure 36. Nomogram for the traditional machine learning models constructed based on CT for diagnosing osteoporosis


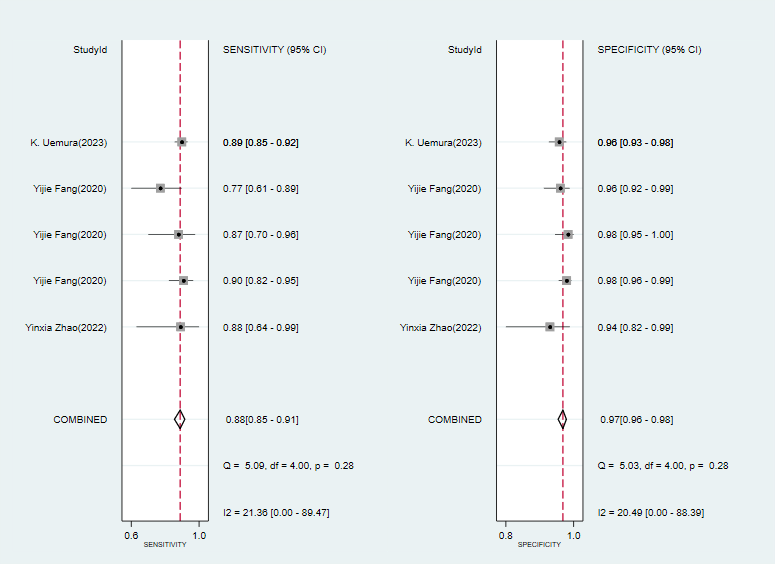


Supplementary Figure 37. Forest plot of sensitivity and specificity for CT-based models based on external validation


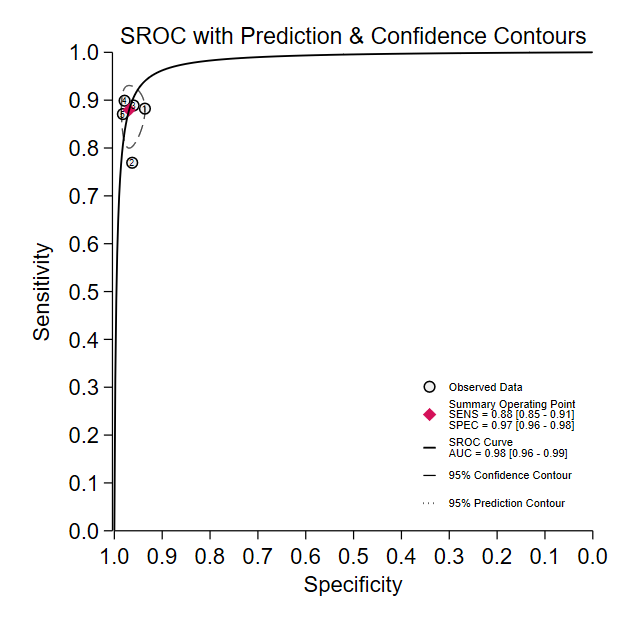


Supplementary Figure 38. SROC curve for CT-based models based on K-fold cross-validation


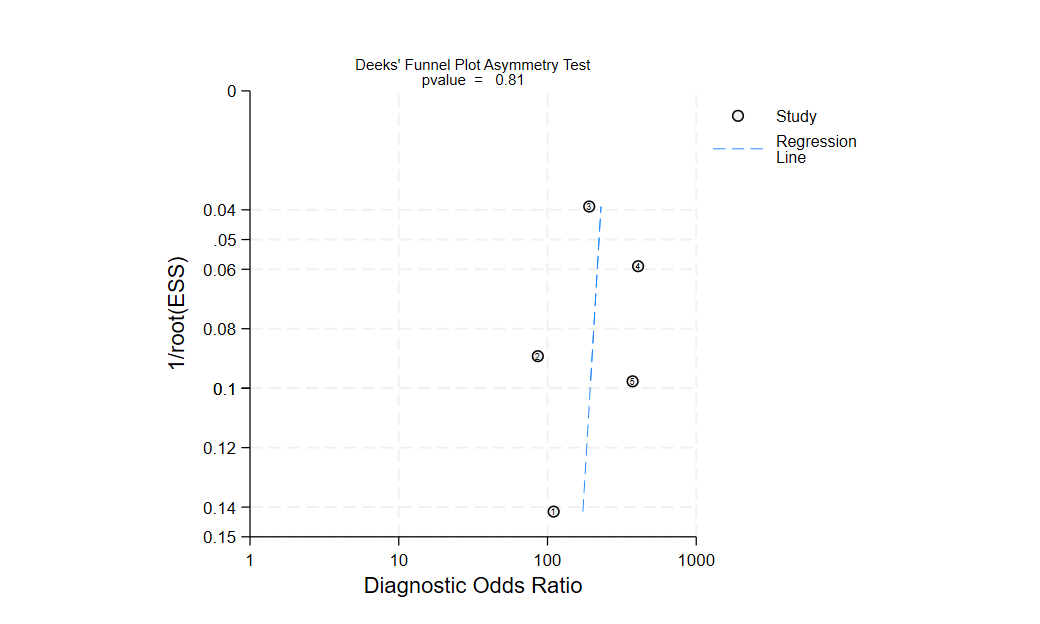


Supplementary Figure 39. Deek’s funnel plot for CT-based models based on K-fold cross-validation


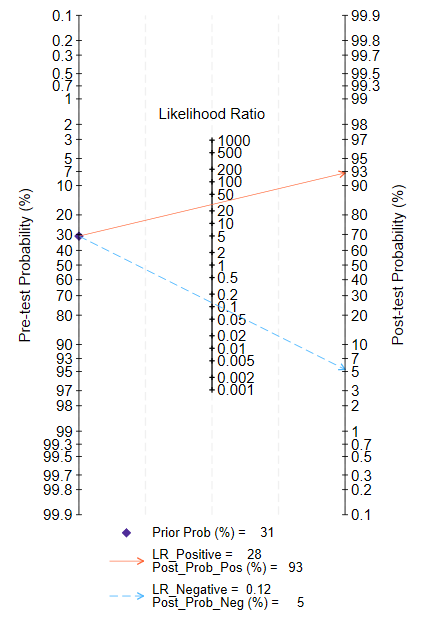


Supplementary Figure 40. Nomogram for CT-based models based on K-fold cross-validation


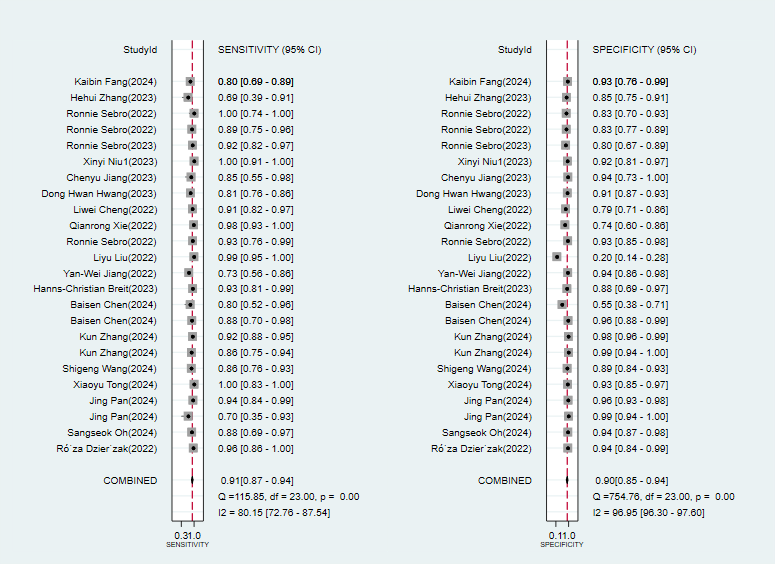


Supplementary Figure 41. Forest plot of sensitivity and specificity for CT-based models based on random sampling


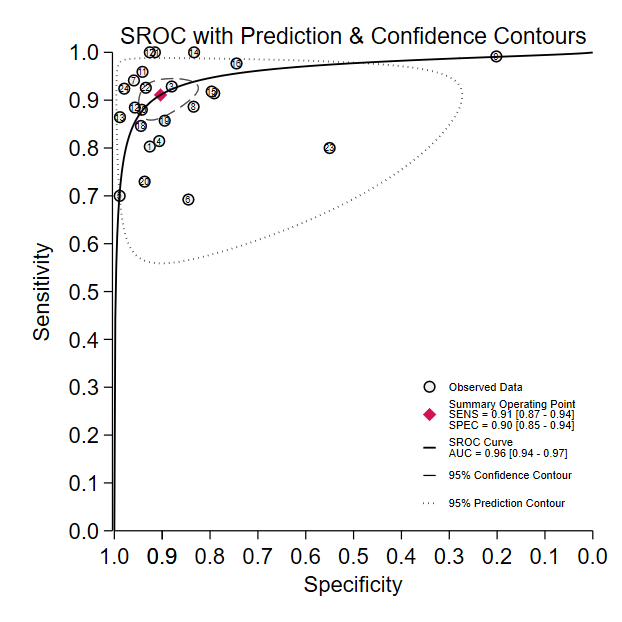


Supplementary Figure 42. SROC curve for CT-based models based on random sampling


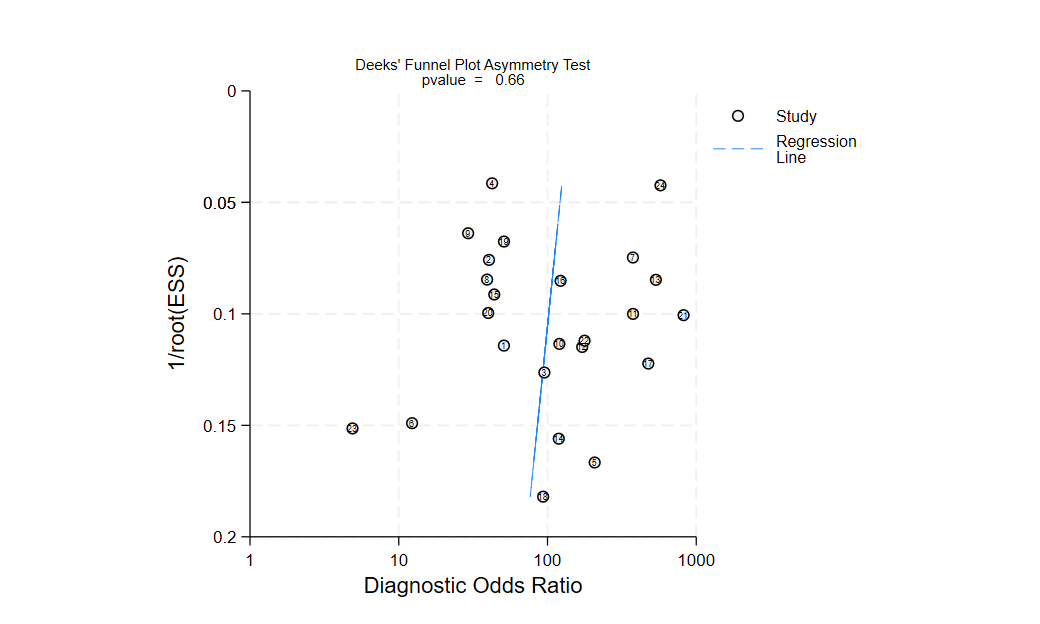


Supplementary Figure 43. Deek’s funnel plot for CT-based models based on random sampling


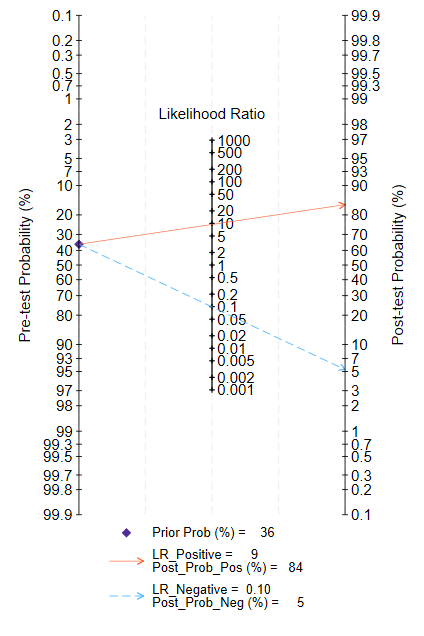


Supplementary Figure 44. Nomogram for CT-based models based on random sampling


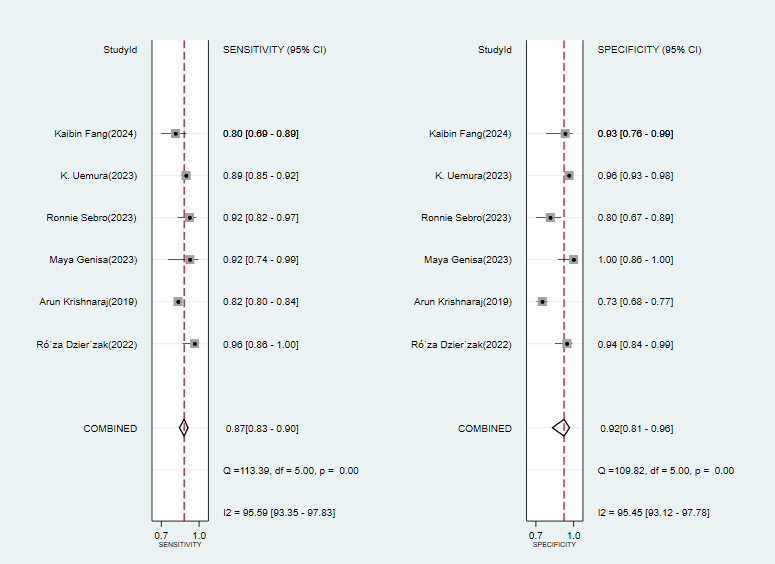


Supplementary Figure 45. Forest plot of sensitivity and specificity of CT-based models for hip joint


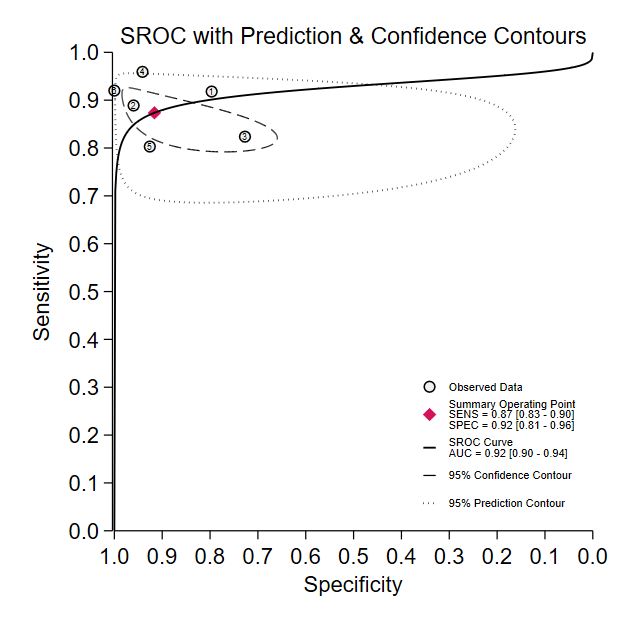


Supplementary Figure 46. SROC curve of CT-based models for hip joint


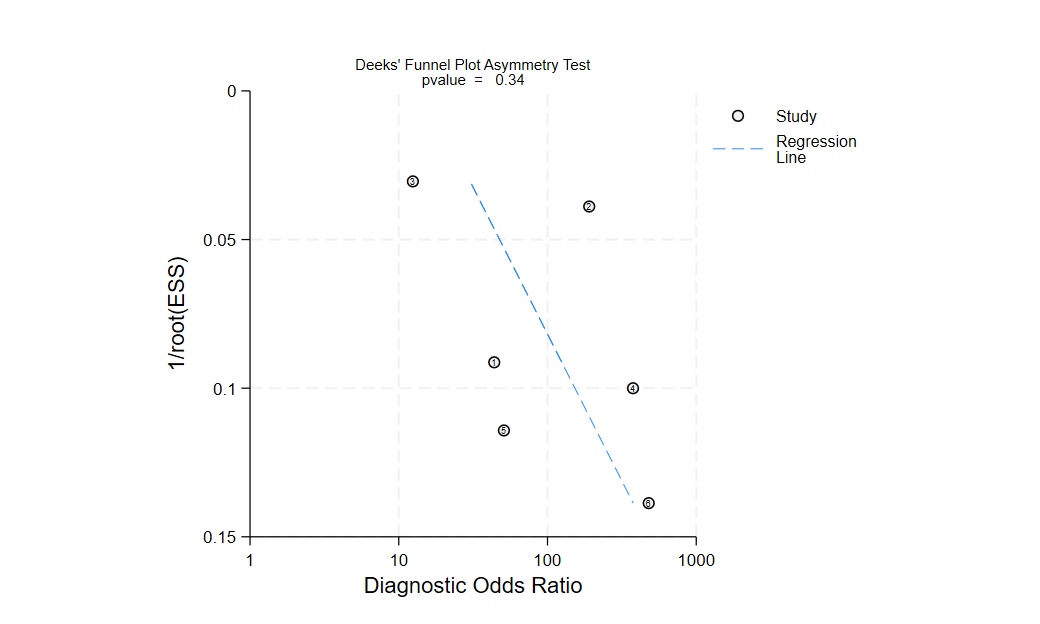


Supplementary Figure 47. Deek’s funnel plot of CT-based models for hip joint


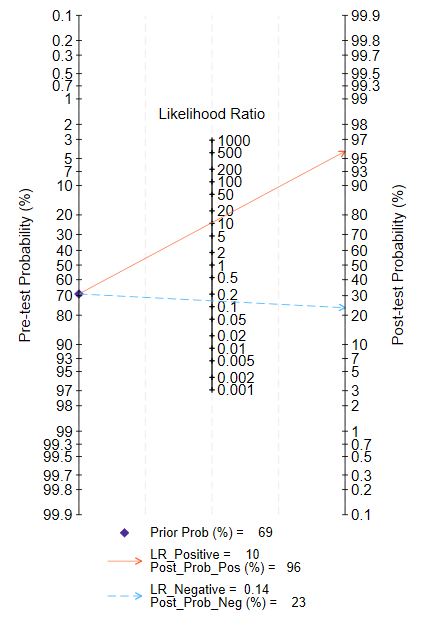


Supplementary Figure 48. Nomogram of CT-based models for hip joint


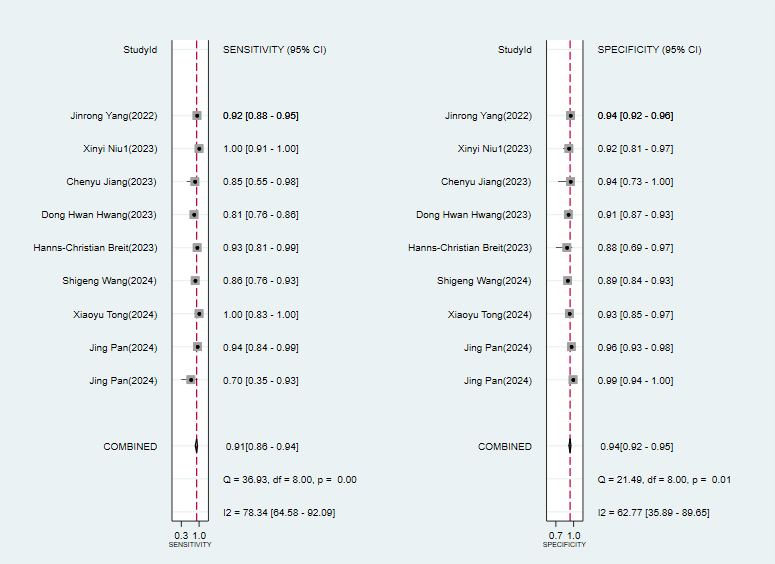


Supplementary Figure 49. Forest plot of sensitivity and specificity of CT-based models for thoracic vertebrae


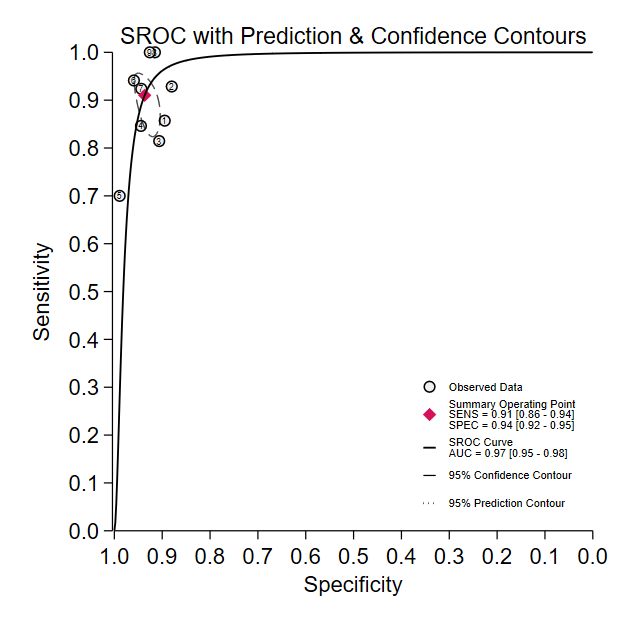


Supplementary Figure 50. SROC curve of CT-based models for thoracic vertebrae


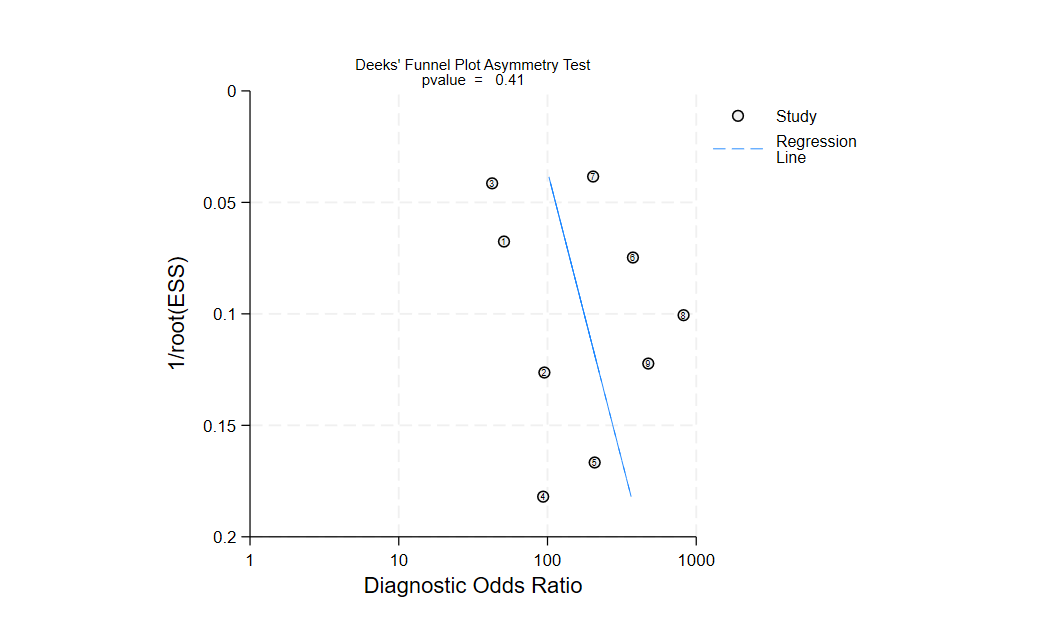


Supplementary Figure 51. Deek’s funnel plot of CT-based models for thoracic vertebrae


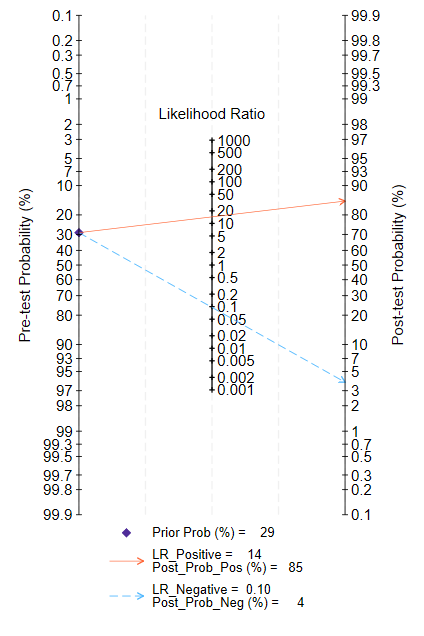


Supplementary Figure 52. Nomogram of CT-based models for thoracic vertebrae


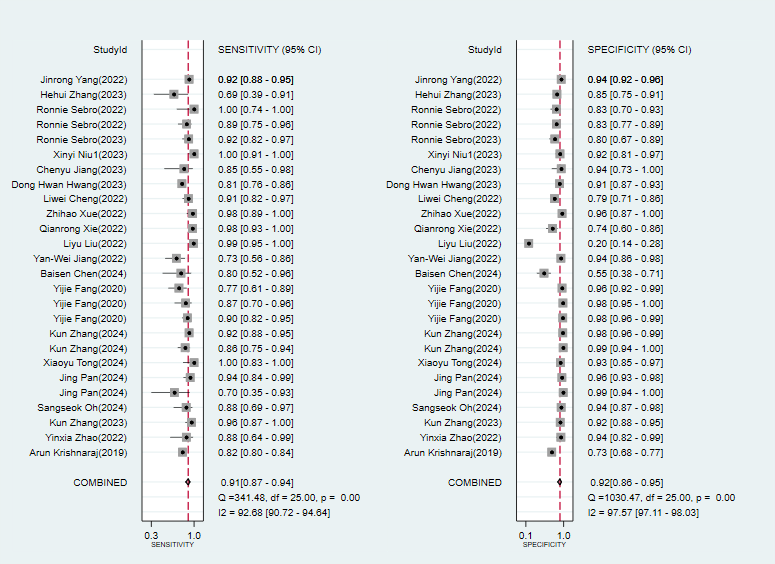


Supplementary Figure 53. Forest plot of sensitivity and specificity of CT-based models for lumbar vertebrae


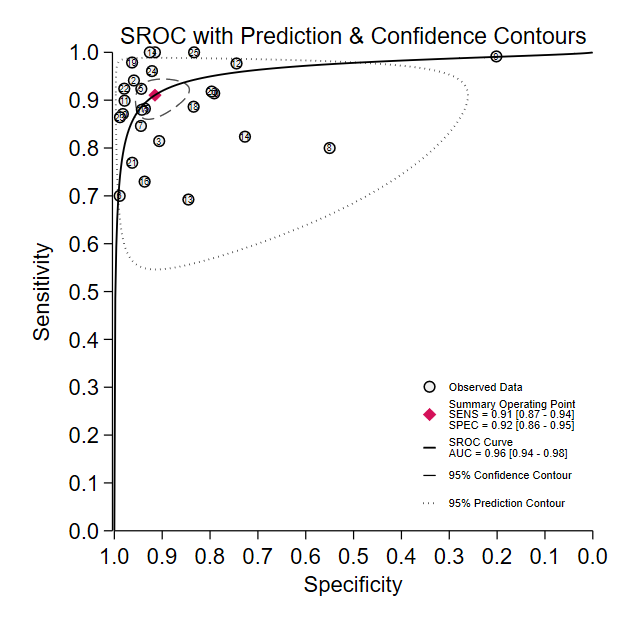


Supplementary Figure 54. SROC curve of CT-based models for lumbar vertebrae


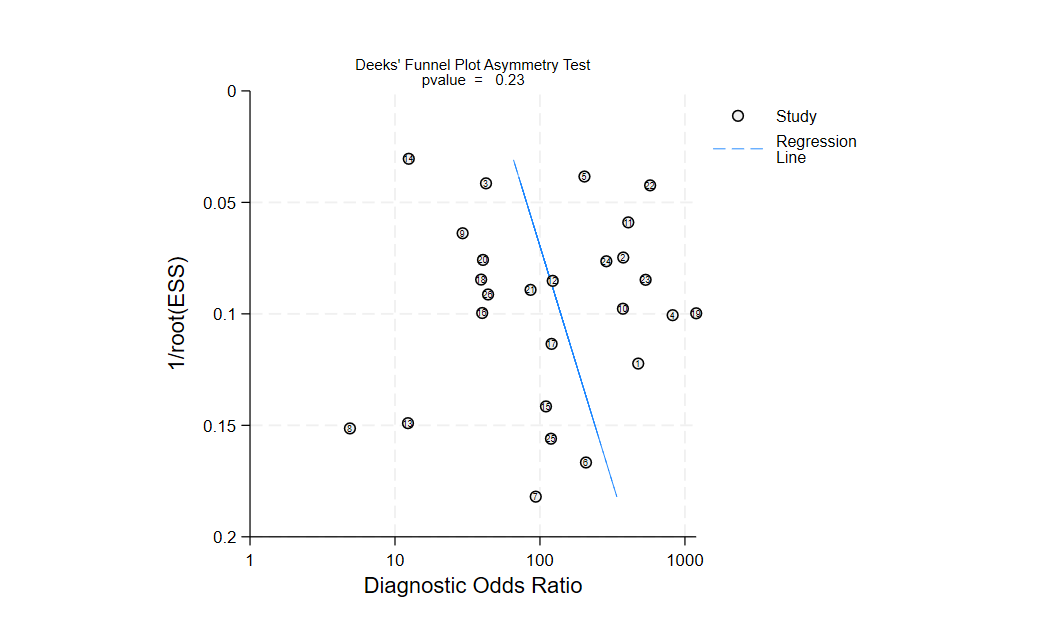


Supplementary Figure 55. Deek’s funnel plot of CT-based models for lumbar vertebrae


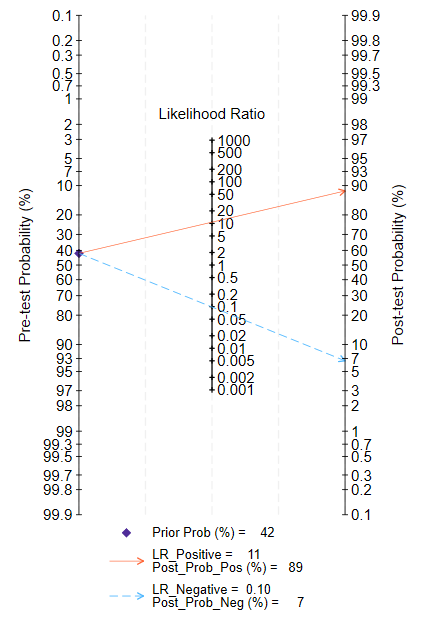


Supplementary Figure 56. Nomogram of CT-based models for lumbar vertebrae
